# Impact of early adverse life events and sex on functional brain networks in patients with urological chronic pelvic pain syndrome (UCPPS): A MAPP Research Network study

**DOI:** 10.1371/journal.pone.0217610

**Published:** 2019-06-20

**Authors:** Arpana Gupta, Ravi R. Bhatt, Bruce D. Naliboff, Jason J. Kutch, Jennifer S. Labus, Priten P. Vora, Mher Alaverdyan, Andrew Schrepf, Susan Lutgendorf, Emeran A. Mayer, MAPP Research Network

**Affiliations:** 1 G. Oppenheimer Center for Neurobiology of Stress and Resilience, UCLA, Los Angeles, CA, United States of America; 2 David Geffen School of Medicine, UCLA, Los Angeles, CA, United States of America; 3 Vatche and Tamar Manoukian Division of Digestive Diseases, UCLA, Los Angeles, CA, United States of America; 4 USC Division of Biokinesiology and Physical Therapy, Los Angeles, CA, United States of America; 5 Chronic Pain and Fatigue Research Center, University of Michigan, Ann Arbor, MI, United States of America; 6 Department of Anesthesiology, University of Michigan, Ann Arbor, MI, United States of America; 7 Department of Psychological and Brain Sciences, University of Iowa, Iowa City, IA, United States of America; 8 Department of Urology, University of Iowa, Iowa City, IA, United States of America; 9 Department of Obstetrics and Gynecology, University of Iowa, Iowa City, IA, United States of America; 10 Ahmanson-Lovelace Brain Mapping Center, UCLA, Los Angeles, CA, United States of America; Florida State University, UNITED STATES

## Abstract

Pain is a highly complex and individualized experience with biopsychosocial components. Neuroimaging research has shown evidence of the involvement of the central nervous system in the development and maintenance of chronic pain conditions, including urological chronic pelvic pain syndrome (UCPPS). Furthermore, a history of early adverse life events (EALs) has been shown to adversely impact symptoms throughout childhood and into adulthood. However, to date, the role of EAL’s in the central processes of chronic pain have not been adequately investigated. We studied 85 patients (56 females) with UCPPS along with 86 healthy controls (HCs) who had resting-state magnetic resonance imaging scans (59 females), and data on EALs as a part of the Multidisciplinary Approach to the Study of Chronic Pelvic Pain (MAPP) Research Network Study. We used graph theory methods in order to investigate the impact of EALs on measures of centrality, which characterize information flow, communication, influence, and integration in a priori selected regions of interest. Patients with UCPPS exhibited lower centrality in the right anterior insula compared to HCs, a key node in the salience network. Males with UCPPS exhibited lower centrality in the right anterior insula compared the HC males. Females with UCPPS exhibited greater centrality in the right caudate nucleus and left angular gyrus compared to HC females. Males with UCPPS exhibited lower centrality in the left posterior cingulate, angular gyrus, middle temporal gyrus, and superior temporal sulcus, but greater centrality in the precuneus and anterior mid-cingulate cortex (aMCC) compared to females with UCPPS. Higher reports of EALs was associated with greater centrality in the left precuneus and left aMCC in females with UCPPS. This study provides evidence for disease and sex-related alterations in the default mode, salience, and basal ganglia networks in patients with UCPPS, which are moderated by EALs, and associated with clinical symptoms and quality of life (QoL).

## 1. Introduction

Urologic Chronic Pelvic Pain Syndrome (UCPPS) is used to describe idiopathic chronic pelvic pain of urologic origin [1], including interstitial cystitis/bladder pain syndrome (IC/BPS), primarily diagnosed in women [2] and chronic prostatitis/chronic pelvic pain syndrome (CP/CPPS), a diagnosis exclusive to men [3]. The hallmark symptom is chronic pain in the pelvic region, urogenital floor or external genitalia, along with symptoms of urinary urgency and frequency [4,5]. UCPPS affects between 1.8% and 26.6% of the population [6,7], and it is thus a major healthcare problem with social and economic consequences [8,9]. However, despite various efforts directed towards identifying the underlying pathophysiology of UCPPS, our current understanding of the syndrome and effectiveness of available treatments remains limited [10]. One of the goals of the Multidisciplinary Approach to the Study of Chronic Pelvic Pain (MAPP) Research Network, a multi-site project funded by the National Institute of Diabetes and Digestive and Kidney Diseases [10,11], is to identify alterations in brain networks in affected patients which may play a role in symptom generation.

Multimodal neuroimaging has characterized abnormalities present in patients with UCPPS [12–18]. As part of the neuroimaging efforts of the MAPP Research Network—which was developed to investigate the underlying pathology of UCPPS and inform clinical management of patients—[1,10,11], alterations in gray matter (GM), white matter (WM) microstructure and resting-state functional connectivity (RS-FC) have been identified in key regions of the sensorimotor, basal ganglia, executive control, emotion regulation, salience, and default mode networks (DMN) [12,15–24]. Alterations in these networks have been associated with behavioral phenotypes, greater pain, anxiety and urinary urgency [14], suggesting they may play an important role in symptom generation, maintenance [25,26] and may be critical targets for therapeutic control of UCPPS.

Attention has also been directed to the influence of psychosocial variables in patients with chronic pain, such as early adverse life events (EALs) during childhood [27–29]. The experience of EALs may result in epigenetic changes within central stress circuits, causing changes through adulthood [30,31], that promote the development of diseases by altering neurodevelopment of myelination, neurogenesis, and synaptic branching [32,33]. A history of EALs has been associated with brain alterations related to the sensorimotor, basal ganglia, emotion regulation, salience, and default mode networks [34–36]. These associations are consistent with the hypothesis that EAL-related brain changes can contribute to UCPPS and its associated symptoms [28,36–39]. Given the relevance of EALs in chronic pain and the brain architecture [27,35], and the lack of incorporation of EALs in current research with patients with UCPPS, we sought to explore how EALs may influence brain phenotypes exhibiting both disease and sex-related differences in UCPPS patients compared to healthy controls.

Graph theory was used to investigate the main disease and sex-related differences of resting-state functional connectivity in patients with UCPPS compared to HCs, as well as the moderating role of EALs on those differences. This analysis method assesses the role of specific brain regions in the functional integrity and information flow (indexed as centrality) within a priori regions from specific brain networks [40–42]. We aimed to test the general hypothesis that a history of EALs is associated with alterations of brain networks, and that these associations differ depending on disease and sex. Furthermore, based on past brain imaging research in chronic pain and UCPPS [13,18,21,43–46], we hypothesized that a history of EALs will moderate: (1) Disease-related differences in measures of centrality to a greater extent in patients with UCPPS compared to non-UCPPS controls in regions of the sensorimotor, basal ganglia, executive control, emotion regulation, salience, and default mode networks, and these alterations will be associated with greater symptom and pain severity and decreased quality of life; and (2) Sex-related differences in measures of centrality to a greater extent in female patients with UCPPS compared to male patients with UCPPS in regions of the DMN, salience, and sensorimotor networks, and these alterations will be associated with greater symptom and pain severity and decreased quality of life.

## 2. Methods

### 2.1. Subjects

Participants included men and women diagnosed with UCPPS and healthy controls (HC). Each participant underwent multimodal neuroimaging phenotyping with structural and resting state scans collected as a part of the Multidisciplinary Approach to the Study of Chronic Pain (MAPP) multisite neuroimaging project [1,11]. Data were collected using standardized acquisition protocols at the various sites. Data were collected using standardized acquisition protocols at the various sites. All subjects provided informed written consent to participate in the current study according to the Declaration of Helsinki. All consenting procedures and protocols were approved by the institutional review board at each of the participating sites, which included University of California Los Angeles, University of Michigan, Stanford University, University of Alabama, Birmingham, and Northwestern University. Detailed procedures, acquisition protocols and a description of the MAPP repository are available at https://repository.niddk.nih.gov/studies/mapp and **S1 Table** [11]. All subjects were asked to keep their eyes closed and not fall asleep during the resting-state scan. Data on some of these subjects has previously been published [12–18,22]. The final data included 85 patients with UCPPS (29 males and 56 females), and 86 healthy control subjects (29 males and 59 females).

### 2.2. Clinical measures

All participants completed the child version of the Childhood Traumatic Events Scale (CTES) [47]. The CTES is a validated and reliable brief survey measuring 6 types of early traumatic experiences during childhood (death, divorce, violence, sexual abuse, illness, or other). Finals scores were composed of the sum of all the endorsed categories. Higher scores reflect greater exposure to traumatic events up to age 17.

Additionally, all participants completed the National Institute of Health’s Genitourinary Pain Index (GUPI). The GUPI is a 9-item instrument developed from the NIH-Chronic Prostatitis Symptom Index (CPSI) [48]. The GUPI versions used by MAPP Research Network include several new items about bladder-specific pain, and male gender-specific items were replaced with female-gender specific items for a women’s version. This revised GUPI is therefore applicable to men and women to assess pain symptoms (0–23 scale), urinary symptoms (0–10 scale), and quality of life (0–12 scale) as separate sub-scales, and overall as a total score [49]. The GUPI assesses symptoms over the past week and was given on the same day as the MRI. It has been shown to be valid, reliable, and responsive to change [49]. Higher scores reflect greater symptom burden and impact on quality of life.

### 2.3. Magnetic resonance imaging: Preprocessing/quality control

A series of standard processes were used to convert the raw neuroimaging data to data ready for statistical analysis. Briefly, after acquiring the raw data, images were preprocessed to deal with timing issues (slice-timing correction), head movement (motion correction), anatomical alignment of various types of scans (co-registration), and transforming the images onto a standard anatomical reference space (spatial normalization or warping). The normalized, co-registered images were then parcellated into regions based on well-regarded atlases, which are used as regions of interest (ROIs) to calculate functional network metrics using graph theory methods.

Structural images were included based on compliance with the acquisition protocol, full brain coverage, minimal motion (< 2 mm in all directions), absence of flow/zipper, and minor atrophy/vascular degeneration. Functional images were included based on compliance with acquisition protocol, full brain coverage, motion estimate of <1/2 voxel size between adjacent time points (keeping a stringent standard across time series for all voxels), ghosting in cerebrum, minimal physiological noise (> 0.2 Hz in frequency spectrum), and few to no outlier voxels, mean intensity shifts, or K-space “spikes.”

### 2.4. Magnetic resonance imaging: Structural brain parcellation

Segmentation and regional parcellation of gray matter images were performed using FreeSurfer [50–52] and in-house workflow pipelines using the Destrieux atlas and the Harvard-Oxford atlas [53]. This parcellation yielded 74 cortical structures, 7 subcortical structures, and the cerebellum for each hemisphere (left and right), plus the brainstem, for a complete set of 165 parcellations for the entire brain.

### 2.5. Magnetic resonance imaging: Resting state functional connectivity

Resting state processing was conducted using SPM8 software (Welcome Department of Cognitive Neurology, London, UK). The first two volumes were discarded to allow for stabilization of the magnetic field. Slice timing correction was performed first, followed by rigid six-degree motion-correction realignment. The motion correction parameters in each degree were examined for excessive motion. Mean frame-wise displacement (FD), and again with root mean squared (RMS) realignment estimates were also calculated as robust measures of motion using publicly available MATLAB code from GitHub [54]. These were used to check for the influence of motion in subsequent supplementary analyses as covariates. The resting state images were then co-registered to their respective anatomical T1 images. Each T1 image was then segmented and normalized to a smoothed template brain in Montreal Neurological Institute (MNI) template space. Each subject's T1 normalization parameters were then applied to that subject's resting state image, resulting in an MNI space normalized resting state image. The resulting images were smoothed with 4mm^3^ Gaussian kernel.

### 2.6. Magnetic resonance imaging: Regions of interest

**Fig 1:**
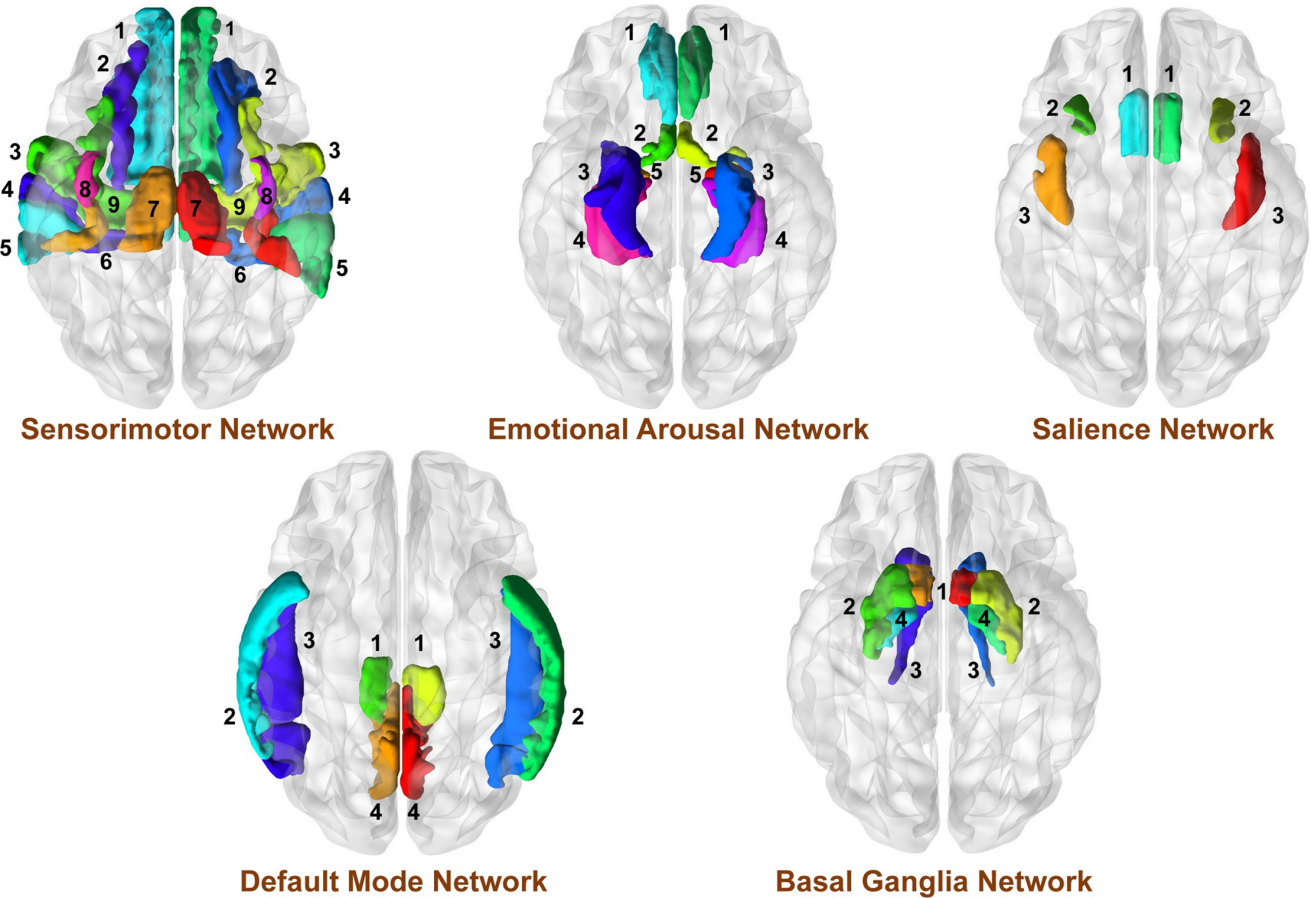
A Priori Regions of Interest (ROIs) selected for analyses. A priori regions of interest (ROIs) are shown along with their Destreuix Atlas labels and Destreuix Atlas short names. Sensorimotor Network: SupFG (1): Superior Frontal Gyrus; SupFS (2): Superior Frontal Sulcus; SupCirIns (3): Superior segment of the circular sulcus of the insula; PRCG (4): Precentral Gyrus; PosCG (5): Postcentral gyrus; PosCS (6): Postcentral Sulcus; PosLS (7): Posterior ramus (or segment) of the lateral sulcus (or fissure); LoInG/Cins (8): Long insular gyrus and central sulcus of the insula; SupPrCs (9): Superior part of precentral sulcus; InfPrCs (10): Inferior part of precentral sulcus; Thal (11): Thalamus Basal Ganglia Network: Nacc (1): Nucleus Accumbens; Put (2): Putamen; CaN (3): Caudate Nucleus; Pal (4): Pallidum/Globus Pallidus Executive Control Network: SupFG (1): Superior Frontal Gyrus; InfFGTrip (2): Triangular Part of the Inferior Frontal Gyrus; InfFS (3): Inferior Frontal Sulcus; SupPL (4): Superior Parietal Lobule Emotion Regulation Network: pgACC (1): Pregenual Anterior Cingulate; sgACC (2): Subgenual Anterior Cingulate; PaHipG (3): Parahippocampal Gyrus; Hipp (4): Hippocampus; Amg (5): Amygdala Salience Network: aMCC (1): Anterior Mid Cingulate Cortex; ACirIns (2): Anterior segment of the circular sulcus of the insula; InfCirIns (3): Inferior segment of the circular sulcus of the insula Default Mode Network: PoPl (1): Planum Polare of the superior frontal gyrus; SupTGLp (2): Lateral aspect of the superior temporal gyrus; MTG (3): Middle Temporal Gyrus; SupTS (4): Superior Temporal Sulcus; AngG (5): Angular gyrus; PrCun (6): Precuneus; PosVCgG (7): Posterior-ventral part of the cingulate gyrus; PosDCgG (8): Posterior-dorsal part of the cingulate gyrus; CGSMarp (9): Marginal branch (or part) of the Cingulate Sulcus.

ROIs were selected based on prior neuroimaging research on patients with UCPPS and with studies related to EALs [15,16,34–36,55,56]. Parcellated regions from the Destrieux atlas and regions of interest evaluated in this study were taken from the original 165 parcellated regions based on previous research and involved the following networks: sensorimotor network (precentral gyrus and sulcus [M1], supplementary motor area [SMA/M2], precentral gyrus and sulcus [S1], thalamus, posterior insula [pINS]) [15,16,18–20,55,57,58]; basal ganglia network (nucleus accumbens [NAcc], putamen [Pu], pallidum [Pal] and caudate nucleus [CaN]) [17,18,20]; executive-control network (ventrolateral prefrontal cortex [vlPFC], dorsolateral prefrontal cortex [dlPFC], posterior parietal cortex [PPC]) [15,17,18,20,22]; emotion regulation network (pregenual anterior cingulate [pgACC], subgenual anterior cingulate [sgACC], parahippocampal gyrus, amygdala [Amg] and hippocampus) [12,16–18,22–24,55]; salience network (anterior insula [aINS], anterior midcingulate cortex [aMCC], orbitofrontal cortex [OFC]) [16,17,20,23,24,55]; and default mode network (precuneus, middle temporal gyrus [MTG], superior temporal gyrus [SupTG], superior temporal sulci [STS], angular gyrus [AngG], posterior cingulate cortex [PCC]) [15–18,20,22,55,56] (See **Table 1** and **Fig 1**). The anterior part of the default mode network was not included in the analysis because of the lack of results regarding this region in past brain imaging studies in patients with UCPPS.

**Table 1:** A priori regions of interest (ROIs) selected for analyses.

Region	Full Name	Label	Reference
**Sensorimotor Network**			
Precentral Gyrus (Primary Motor Cortex, M1)	Precentral gyrus	PRCG	[12,16,18,20–22]
Precentral Sulcus (Primary Motor Cortex, M1)	Superior part of the precentral sulcus	SupPrCs	[12,16,18,20–22]
	Inferior Part of the precentral sulcus	InfPrCS	[12,16,18,20–22]
Postcentral Gyrus (Primary Somatosensory Cortex, S1)	Postcentral Gyrus	PosCG	[12,14,15,18,21]
Postcentral Sulcus (Primary Somatosensory Cortex, S1)	Postcentral Sulcus	PosCS	[12,14,20]
Posterior insula (pINS)	Long insular gyrus and central sulcus of the insula	LoInG/CInS	[15,16,23]
	Posterior ramus (or segment) of the lateral sulcus (or fissure)	PosLS	[15,16,23]
	Superior segment of the circular sulcus of the insula	SupCirInS	[15,16,23]
Supplementary Motor Area (SMA/Secondary Motor Cortex/M2)	BA6/Superior Frontal Gyrus	SupFG	[12,14–16,21,24]
	BA6/Superior Frontal Sulcus	SupFS	[12,14–16,21,24]
Thalamus	Thalamus	Thal	[17,18,22,24]
**Basal Ganglia Network**			
Nucleus Accumbens (NAcc)	Nucleus Accumbens	Nacc	[18,20]
Putamen	Putamen	Pu	[17,18,20]
Pallidum/Globus Pallidus	Pallidum	Pal	[17,18]
Caudate Nucleus	Caudate Nucleus	CaN	[18]
**Executive Control Network**			
Ventrolateral Prefrontal Cortex	Inferior Frontal Sulcus	InfFS	[20,22]
	Triangular Part of the Inferior Frontal Gyrus	InfFGTrip	[22]
Dorsolateral Prefrontal Cortex	Superior Frontal Gyrus	SupFG	[20]
Posterior Parietal Cortex	Superior Parietal Lobule	SupPL	[14,15,17,20]
**Emotion Regulation Network**			
Pregenual Anterior Cingulate (pgACC)	Anterior part of the cingulate gyrus and sulcus	ACgG/S	[17,18,20]
Subgenual Anterior Cingulate (sgACC)	Subcallosal area, subcallosal gyrus	SbCag	[17,18]
Parahippocampal Gyrus	Parahippocampal gyrus, parahippocampal part of the medial occipito-temporal gyrus	PaHipG	[20,22,23]
Hippocampus	Hippocampus	Hip	[12,17,20]
Amygdala	Amygdala	Amg	[12,17,23,24]
**Salience Network**			
Anterior Insula (aINS)	Inferior segment of the circular sulcus of the insula	InfCirIns	[17,21,24]
	Anterior segment of the circular sulcus of the insula	ACirInS	[17,21,24]
Anterior Mid-Cingulate Cortex (aMCC)	Anterior Midcingulate Gyrus	MACgG_S	[21,23,24]
Orbitofrontal Cortex	Suborbital Sulcus	SbOrS	[17,20]
**Default Mode Network**			
Precuneus	Precuneus	PrCun	[14,15,17,22]
	Marginal branch (or part) of the Cingulate Sulcus	CgSMarp	[17]
Angular Gyrus	Angular Gyrus	AngG	[17]
Middle Temporal Gyrus	Middle Temporal Gyrus	MTG	[20]
Superior Temporal Gyrus	Lateral aspect of the superior temporal gyrus	SupTGLp	[16]
	Planum polare of the superior temporal gyrus	PoPl	[16]
Superior Temporal Sulci	Superior Temporal Sulci	SupTS	[16,20]
Posterior Cingulate Cortex	Posterior-dorsal part of the cingulate gyrus (dPCC)	PosDCgG	[17,20]
	Posterior ventral part of the cingulate gyrus (vPCC)	PosVCgG	[17]

A priori regions of interest (ROIs) are shown along with their Destreuix Atlas labels and Destreuix Atlas short names. Sensorimotor Network: SupFG (1): Superior Frontal Gyrus; SupFS (2): Superior Frontal Sulcus; PRCG (3): Precentral Gyrus; SupPrCS/InfPrCS (4): Precentral Sulcus; PosCG (5): Postcentral Gyrus; PosCS (6): Postcentral Sulcus; SuMarG (7): Supramarginal Gyrus; PosLS (8): Posterior ramus (or segment) of the lateral sulcus (or fissure); Thal (9): Thalamus; SupCirIns (10): Superior segment of the circular sulcus of the insula; LoInG/Cins (11): Long insular gyrus and central sulcus of the insula. Basal-Ganglia Network: Nacc (1): Nucleus Accumbens; Put (2): Putamen; CaN (3): Caudate Nucleus; Pal (4): Pallidum/Globus Pallidus. Emotion Regulation Network: pgACC (1): Pregenual Anterior Cingulate; sgACC (2): Subgenual Anterior Cingulate; PaHipG (3): Parahippocampal Gyrus; Hipp (4): Hippocampus; Amg (5): Amygdala. Executive Control Network: InfFS (1): Inferior Frontal Sulcus; InfFGTrip (2): Triangular Part of the Inferior Frontal Gyrus; SupFG (3): Superior Frontal Gyrus; SupPL (4): Superior Parietal Lobule. Salience Network: aMCC (1): Anterior Mid Cingulate Cortex; ACirIns (2): Anterior segment of the circular sulcus of the insula; InfCirIns (3): Inferior segment of the circular sulcus of the insula. Default Mode Network: CGSMarp (1): Marginal branch (or part) of the Cingulate Sulcus; MTG (2): Middle Temporal Gyrus; SupTS (3): Superior Temporal Sulcus; (4): Superior Temporal Gyrus (SupTGLp, PoPl); PrCun (5): Precuneus; (6): Posterior Cingulate Cortex (PCC); (7): Angular Gyrus (AngG).

### 2.7. Magnetic resonance imaging: Resting state functional brain network construction

To determine functional connectivity between the parcellated 165 regions, seed-to-seed characterization was performed by uploading the preprocessed and normalized functional images into the CONN-fMRI functional connectivity toolbox version 13 [59] (http://www.nitrc.org/projects/conn). The normalized, co-registered images were further pre-processed and analyzed using the SPM-based CONN toolbox version 13 [59]. Resting-state images were filtered using a band pass filter to reduce the low and high frequency noises. A component-based noise correction method, CompCor [59] was applied to remove nuisances for better specificity and sensitivity. Six motion realignment parameters (3 translation, and 3 rotational), along with confounds for white matter and CSF were removed using regression. The parcellation and the functional connectivity results were combined to produce a 165x165 weighted, undirected connectivity matrix. ROI-to-ROI functional connectivity analysis was performed between all the ROIs in the CONN-fMRI functional connectivity toolbox. Connectivity correlation coefficients representing the association between average temporal BOLD time series signals across all voxels in the brain were calculated using a general linear model. Each region of interest was Fisher’s r to z transformed, and bivariate correlation maps were smoothed with a 4 mm isotropic Gaussian kernel and submitted into group-level analyses implemented in SPM8. The correlation coefficients from the CONN-fMRI functional connectivity toolbox are thresholded at Z>0.3 and all other values are set to 0. We did not use a proportional-based thresholding approach as it’s been shown that in patient vs. control studies a minimal difference in overall functional connectivity may introduce group differences in network metrics, and a threshold of 0.3 was chosen since a correlation of 0.3 represents a medium effect size, and the inclusion of lower correlations could result in the inclusion of less accurate estimates [60,61]. This information was then used for subsequent analysis. Furthermore, sensitivity analyses were completed at Z>0.4, 0.5, 0.6, 0.7, and 0.8 to determine robustness of significant results (**S2 Table**).

### 2.8. Magnetic resonance imaging: Computing network metrics

The Graph Theoretic GLM toolbox [62] and in-house MATLAB scripts were used to compute graph theoretical brain network properties representing centrality and organization from the subject-specific functional brain networks. Several local-weighted network metrics indexing centrality were computed. Regions with high centrality are highly influential, communicate with many other regions, facilitate flow of information, and play a key role in network resilience to insult [41]. Specifically, three indices representing centrality were computed: 1) *Strength*: reflecting the weighted version of the number of connections present to a given node, 2) *Betweenness centrality*: reflecting the proportion of shortest paths that go through a given node, and 3) *Eigenvector Centrality*: reflecting a self-referential measure defined as a number highly-connected brain regions to which a given brain region is connected, thus serving as a modulating region.

### 2.9. Magnetic resonance imaging: Network metrics brain data visualization

The network metrics output was then input into in-house MATLAB programs to display significant ROIs for each group and then visualized using BrainNet [63].

### 2.10. Statistical analysis

The primary analyses of the above brain imaging metrics and clinical variables entailed a series of 4 a priori contrasts examining the influence of EALs on disease and sex group-based differences in measures of centrality; 1) UCPPS vs. HC (disease effect), 2) Males with UCPPS vs. HC Males (disease effect within males), 3) Females with UCPPS vs. HC Females (disease effect within females), and 4) Males with UCPPS vs. Females with UCPPS (sex effect). Significance was set at α = .05. Permuted probability values were corrected using an FDR adjusted p-value, where a FDR q<0.05 was considered significant. This correction was performed within each contrast, each measure of centrality, by the number of regions in each network (reward, salience, sensorimotor), and by laterality (left vs. right). Due to multi-site acquisition of imaging data, subject network metric data was first transformed and controlled to account for neuroimaging site differences by subtracting from each measure that subject’s site-specific average and then adding the global average. Additionally, to address the possible effects of motion in the scanner contributing to statistical differences, the aforementioned analyses were run with mean frame-wise displacement (FD), and again with root mean squared (RMS) realignment estimates as covariates (**S3A–S3C Table**) [54].

The brain metrics that showed significant group differences in these contrasts were followed up with a moderation analysis testing if the group difference was altered depending on the level of self-reported EALs (CTES total score). The SPSS PROCESS program was used to determine if EALs were a significant moderator of the group contrast. The PROCESS analysis examined the Johnson-Neyman’s region(s) to determine the significance of the group difference at low, medium and high levels of EALs. All moderation tests were run with two-tailed confidence intervals set at 95% and statistical significance was set to α = 0.05. Age was used as covariate in all the moderation analyses.

To determine the associations between the group-related alterations (either disease and/or sex based) in network metrics and clinical variables, partial correlations were run while controlling for age.

## 3. Results

### 3.1. Demographics and clinical variables

**Fig 2:**
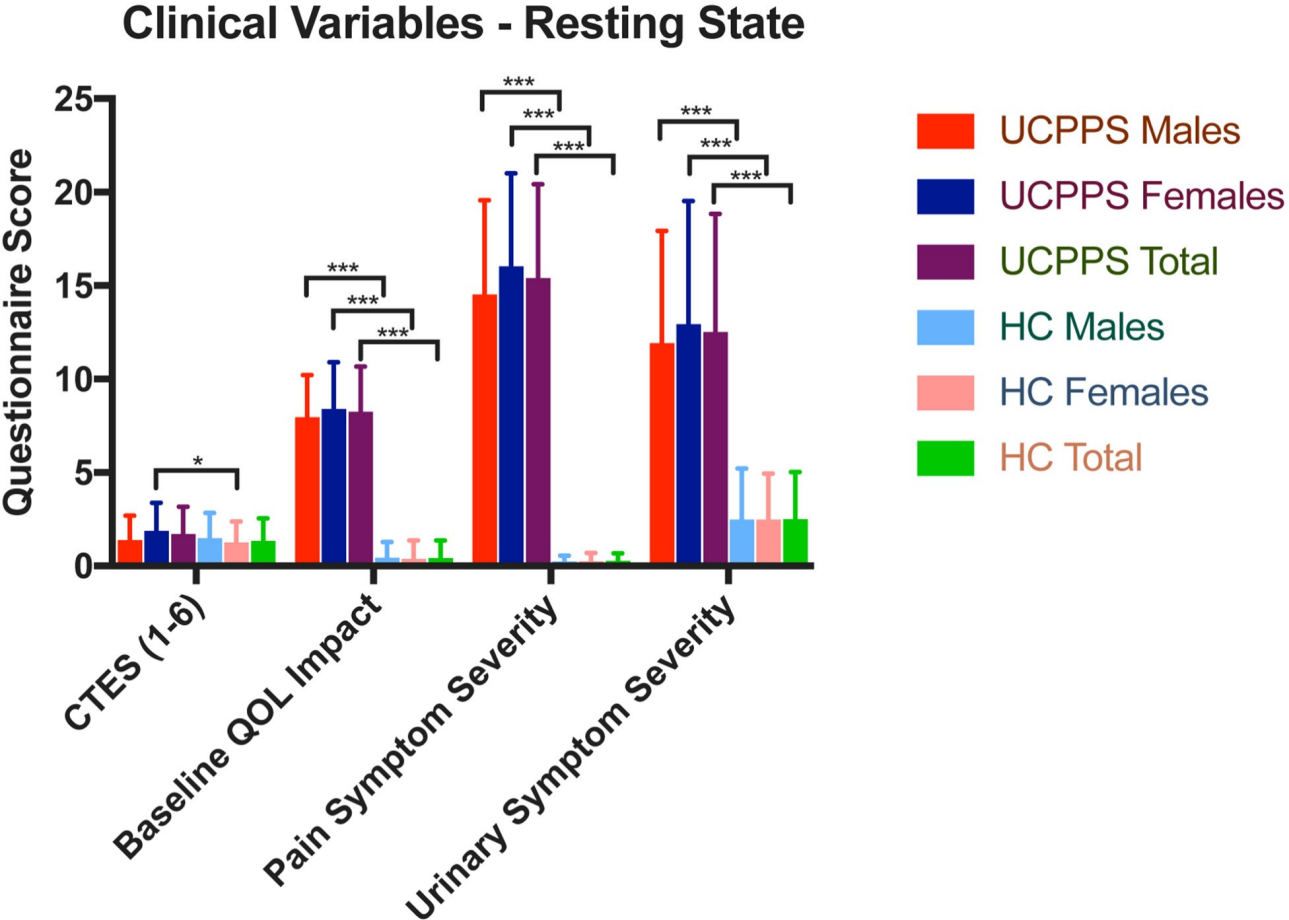
Differences in clinical behavioral measures. Groups: UCPPS: Urological chronic pelvic pain syndrome, HC: Healthy controls Questionnaires: Childhood Traumatic Events Scale (CTES), Baseline GUPI (Genitourinary Pain Index), Pain Severity (GUPI Pain Subscale Score), Urinary Severity (GUPI Urinary Subscale Score). *p < .05, ***p < .001.

There were no significant differences in age between groups for any contrast. Females with UCPPS (mean = 1.89, SD = 1.49, N = 56) had greater CTES scores compared to HC females (mean = 1.25; SD = 1.14; N = 59; F = 6.61, *q* = .02). Patients with UCPPS had lower baseline levels of QoL (F_(1,167)_ = 678.43, *q* = 4.95e-^59^), higher pain severity (F_(1,167)_ = 654.20, *q* = 7.61e-^54^), and urinary severity scores (F_(1,167)_ = 150.10, *q* = 2.82e-^23^) compared to HCs. Both males with UCPPS and females with UCPPS had significantly lower baseline levels of QoL and greater pain and urinary symptom severity compared to HC males and females respectively. (See **Table 2, Table 3** and **Fig 2**).

**Table 2:** Population demographics and clinical/behavioral measures.

	UCPPS												Healthy Controls												Total Subjects			
	Males				Females				Total				Males				Females			Total								
	N = 29				N = 56				N = 85				N = 27				N = 59			N = 86					N = 171			
	Mean	SD	Range	N	Mean	SD	Range	N	Mean	SD	Range	N	Mean	SD	Range	N	Mean	SD	Range	N	Mean	SD	Range	N	Mean	SD	Range	N
Age	40.11	13.71	56.27	29	38.96	12.41	43.86	56	39.36	12.80	57.37	85	49.60	13.55	49.60	27	35.44	10.82	36.49	59	37.90	12.23	52.76	86	38.62	12.50	57.37	171
BMI	26.30	4.09	17.85	29	24.65	5.95	33.64	40	25.34	5.28	33.64	69	14.224	3.75	14.22	25	25.22	5.31	25.40	53	25.17	4.84	25.40	78	25.26	5.04	33.64	147
Early Adversity (CTES)	1.38	1.32	5.00	29	1.89	1.50	6.00	56	1.72	1.45	6.00	85	1.48	1.37	5.00	27	1.25	1.13	5.00	59	1.32	1.21	5.00	86	1.52	1.35	6.00	171
Baseline GUPI QoL Score	7.97	2.24	7.00	29	8.41	2.50	9.0	56	8.26	2.41	9.0	85	0.44	0.85	3.00	27	0.39	0.98	6.00	59	0.41	0.94	6.00	86	4.31	4.34	12.00	171
Pain Severity	14.54	5.04	20.00	29	16.04	4.98	23.0	40	15.41	5.02	24.0	69	0.12	0.44	2.00	25	0.13	0.55	3.00	53	0.13	0.52	3.00	78	7.30	8.39	26.00	147
Urinary Severity	11.93	6.01	21.00	29	12.95	6.58	22.0	40	12.52	6.32	24.0	69	2.48	2.74	9.00	25	2.49	2.44	10.00	53	2.49	2.53	10.00	78	7.20	6.87	24.00	147

Groups: UCPPS: Urological chronic pelvic pain syndrome, HC: Healthy controls. Questionnaires: Body Mass Index (BMI), Childhood Traumatic Events Scale (CTES), Baseline GUPI (Genitourinary Pain Index), Pain Severity (GUPI Pain Subscale Score), Urinary Severity (GUPI Urinary Subscale Score). Abbreviations: Sample size (N), Standard Deviation (SD). Significance: p < .05

**Table 3:** Differences in population demographics and clinical/behavioral measures.

	UCPPS vs. HC			Males with UCPPS vs. Females with UCPPS			Males with UCPPS vs. HC Males			Females with UCPPS vs. HC Females		
Measurement	F-value	p-value	q-value	F-value	p-value	q-value	F-value	p-value	q-value	F-value	p-value	q-value
Age	0.008	0.93	0.95	0.168	0.68	0.82	0.92	0.34	0.36	2.35	0.13	0.16
BMI	0.15	0.70	0.73	1.78	0.19	0.49	0.80	0.37	0.39	0.29	0.59	0.62
Early Adversity (CTES)	1.526	0.22	0.24	2.84	0.09	0.42	0.08	0.78	0.79	6.61	0.01	**0.02**
Baseline GUPI QoL Score	678.431	1.05E-60	**4.95E-59**	1.13	0.29	0.62	236.19	8.72E-34	**4.10E-32**	551.98	8.09E-55	**3.80E-53**
Pain Severity	654.199	3.24E-55	**7.61E-54**	3.20	0.08	0.40	234.94	5.57E-32	**1.31E-30**	485.71	7.86E-48	**1.23E-46**
Urinary Severity	150.096	4.80E-24	**2.82E-23**	0.78	0.38	0.66	53.74	1.55E-11	**1.04E-10**	111.75	1.17E-19	**5.50E-19**

Groups: UCPPS: Urological chronic pelvic pain syndrome, HC: Healthy controls. Questionnaires: Body Mass Index (BMI), Childhood Traumatic Events Scale (CTES), Baseline GUPI (Genitourinary Pain Index), Pain Severity (GUPI Pain Subscale Score), Urinary Severity (GUPI Urinary Subscale Score). Abbreviations: F statistic (F-value), p value (p-value), FDR corrected p value (q-value). Significance: p < .05

### 3.2. Differences in resting-state functional centrality

**Fig 3:**
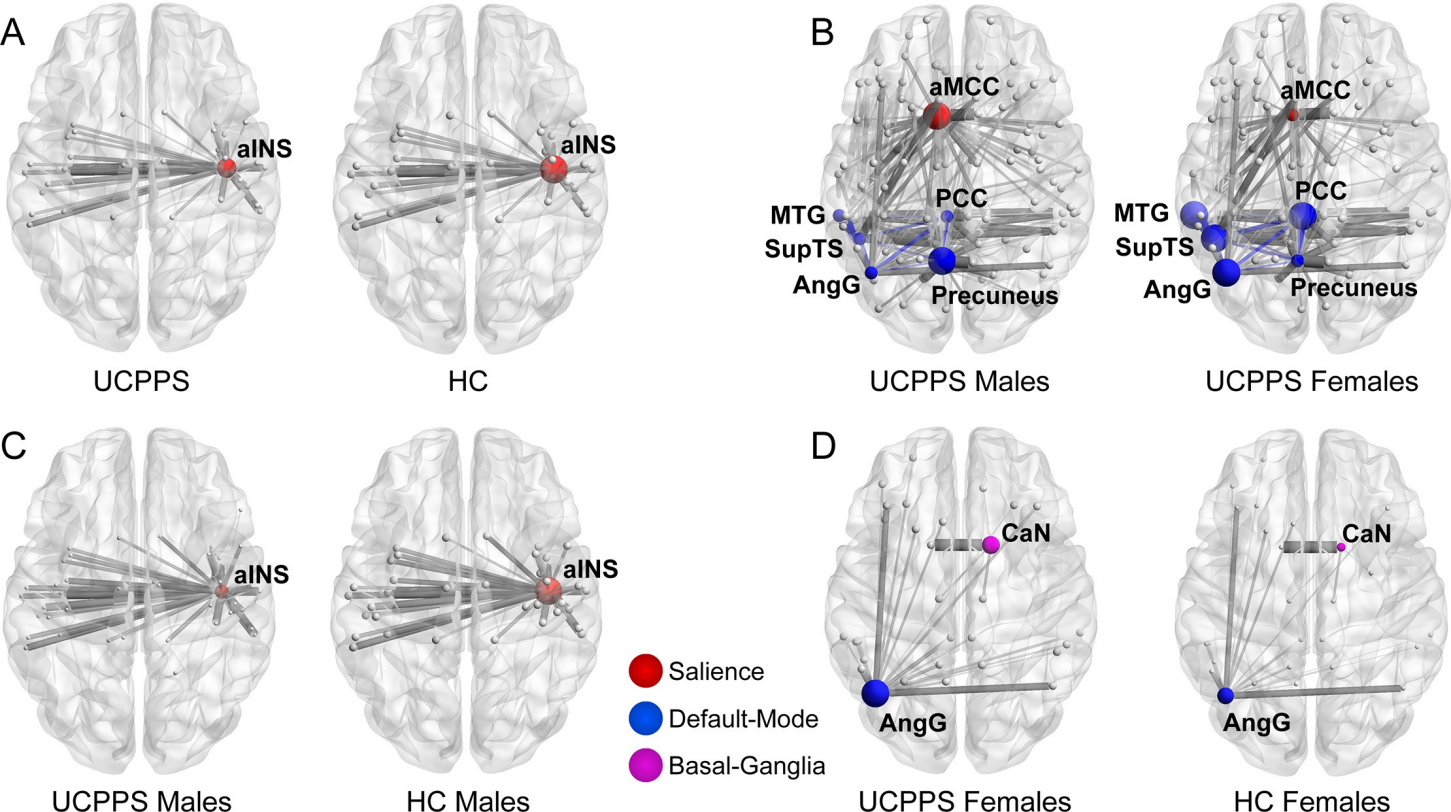
Differences between groups in measures of centrality. UCPPS: Urological Chronic Pelvic Pain Syndrome. Salience Network: aINS: anterior insula, aMCC: anterior mid-cingulate cortex Default Mode Network: AngG: angular gyrus, PCC: posterior cingulate cortex, MTG: middle temporal gyrus, SupTS: superior temporal sulcus, Precuneus: precuneus Basal Ganglia Network: CaN: caudate nucleus.

Results indicating differences in measures of centrality between groups are shown in **Table 4** and **Fig 3**. A summary of the group-based differences has been provided in **Table 5**.

**Table 4:** Significant resting-state functional network metrics.

**UCPPS vs. HC**										
**Network**	**Region of Interest**		**Network Metric**	**t-value**		**p-value**	**q -value**	**B value**	**Cohen's d**	**Interpretation**
**Salience**	Right Anterior Insula (InfCirIns)		Betweeness Centrality	-3.15		0.007	0.030	-44.00	-0.32	UCPPS↓ HC ↑
**Males with UCPPS vs. HC Males**										
**Network**	**Region of Interest**	**Network Metric**			**t-value**	**p-value**	**q -value**	**B value**	**Cohen's d**	**Interpretation**
**Salience**	Right Anterior Insula (InfCirIns)	Eigenvector Centrality			-3.99	0.0003	0.001	-181.47	-0.56	UCPPS M ↓ HC M ↑
**UCPPS Females vs. HC Females**										
**Network**	**Region of Interest**		**Network Metric**		**t-value**	**p-value**	**q -value**	**B value**	**Cohen's d**	**Interpretation**
**Basal Ganglia**	Right Caudate Nucleus		Betweeness Centrality		3.20	0.006	0.023	83.61	0.52	UCPPS F ↑ HC F ↓
**Default Mode**	Left Angular Gyrus		Betweeness Centrality		3.18	0.006	0.037	119.00	0.52	UCPPS F ↑ HC F ↓
**UCPPS Males vs. UCPPS Females**										
**Network**	**Region of Interest**		**Network Metric**		**t-value**	**p-value**	**q -value**	**B value**	**Cohen's d**	**Interpretation**
**Default Mode**	Left Posterior Cingulate Cortex (PosDCgG)		Eigenvector Centrality		-3.10	0.008	0.038	-0.037	-0.52	UCPPS M ↓ UCPPS F ↑
	Left Angular Gyrus		Eigenvector Centrality		-2.81	0.018	0.042	-0.033	-0.64	UCPPS M ↓ UCPPS F ↑
	Left Middle Temporal Gyrus		Eigenvector Centrality		-2.95	0.013	0.038	-0.034	-0.55	UCPPS M ↓ UCPPS F ↑
	Left Superior Temporal Sulcus		Eigenvector Centrality		-2.98	0.012	0.038	-0.034	-0.53	UCPPS M ↓ UCPPS F ↑
	Left Precuneus (CgSMarp)		Eigenvector Centrality		2.73	0.024	0.043	0.029	0.37	UCPPS M ↑ UCPPS F ↓
	Left Precuneus (CgSMarp)		Strength		3.25	0.005	0.045	5.68	0.31	UCPPS M ↑ UCPPS F ↓
**Salience**	Left aMCC		Strength		3.14	0.007	0.030	4.84	0.58	UCPPS M ↑ UCPPS F ↓

Groups: UCPPS: Urological chronic pelvic pain syndrome, HC: Healthy controls. Regions: InfCirIns: Inferior segment of the circular sulcus of the insula, PosDCgG: Posterior dorsal part of the cingulate gyrus, CGSMarp: Marginal branch of the cingulate sulcus, aMCC: Anterior mid-cingulate cortex

↑ = Higher

↓ = Lower

**Table 5:** Summary of network Metrics findings and moderating effects.

Summary Table										
Network	Region of Interest	Contrast	Network Metric	ETI	UCPPS	HC	Males with UCPPS	Females with UCPPS	HC Males	HC Females
**Salience**	Right aINS	UCPPS vs. HC	Betweenness Centrality	↑	↑	↑				
	Right aINS	Males with UCPPS vs. HC Males	Betweenness Centrality	↑			↓		↑	
	Left aMCC	Males with UCPPS vs Females with UCPPS	Strength	↑			↓	↑		
**Default Mode**	Left MTG	Males with UCPPS vs. Females with UCPPS	Eigenvector Centrality	↑			↓	↓		
	Left SupTS	Males with UCPPS vs. Females with UCPPS	Eigenvector Centrality	↑			↓	↑		
	Left Precuneus	Males with UCPPS vs. Females with UCPPS	Eigenvector Centrality	↑			↑	↓		
			Strength	↑			↓	↑		
	Left PCC	Males with UCPPS vs. Females with UCPPS	Eigenvector Centrality	↑			↓	↓		
	Left AngG	Males with UCPPS vs. Females with UCPPS	Eigenvector Centrality	↑			↓	↓		
		UCPPS Females vs. HC Females	Betweenness Centrality	↑				↓		↓
**Basal Ganglia**	Right CaN	UCPPS Females vs. HC Females	Betweenness Centrality	↑				↓		↑

Summary table for network metrics findings by group are summarized along with their moderating effect. Regions: MTG: Middle temporal gyrus, SupTS: Superior Temporal Sulcus, aMCC: anterior mid-cingulate cortex, aINS: anterior insula, CaN: caudate nucleus, AngG: angular gyrus, PCC: posterior cingulate cortex.

↑ = Higher

↓ = Lower

#### 3.2.1. UCPPS vs. HC (disease effect)

**Fig 4:**
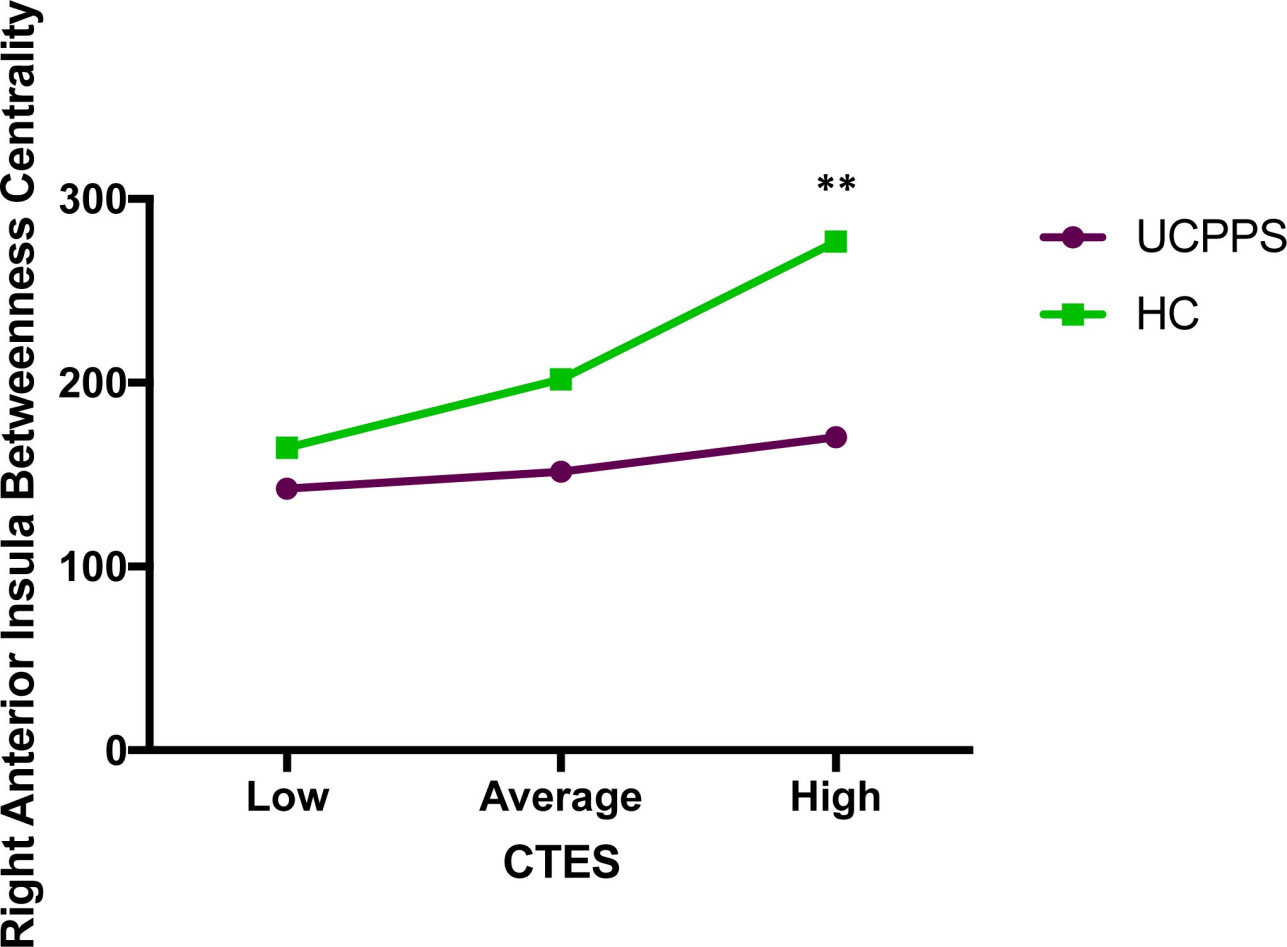
Moderating effect of CTES on the effect of disease on betweenness centrality of the right anterior insula (salience network). Abbreviations: UCPPS: Urologic Chronic Pelvic Pain Syndrome, HC: Healthy Controls, CTES: Childhood Traumatic Events Scale **p = 0.01.

Salience Network: Patients with UCPPS exhibited *lower* betweenness centrality in the right anterior insula compared to HCs (*t* = -3.15, *q* = .03) (**Table 4**, **Table 5** and **Fig 3**). The overall model investigating disease group comparisons predicting right anterior insula betweenness centrality with CTES scores as the moderator, and site and age as covariates, significantly accounted for 8% of the variance (*F*_(5, 156)_ *=* 2.61, *p* = .03, *R*^*2*^ = .08). Investigation at the specific levels of EALs revealed that the moderation took effect at high CTES scores. When compared to patients with UCPPS, HCs had a stronger effect of high CTES scores on betweenness centrality of the right anterior insula (*B =* 106.36, *t*_(156)_ = 2.61, *p* = .01). (**Fig 4**).

#### 3.2.2. Males with UCPPS vs. HC males (disease effect within males)

**Fig 5:**
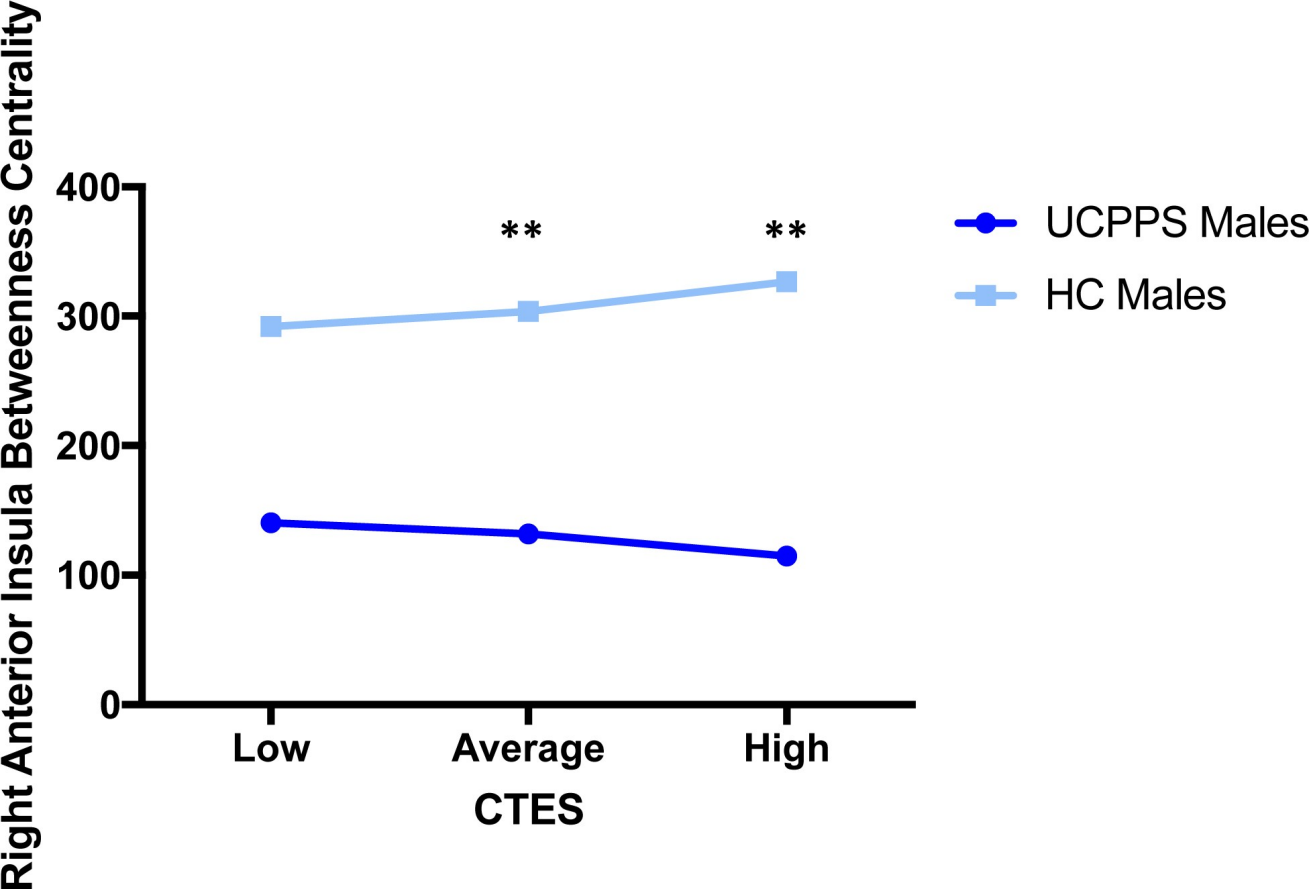
Moderating effect of CTES on the effect of disease within males on centrality of the right anterior insula (salience network). Abbreviations: UCPPS: Urologic Chronic Pelvic Pain Syndrome, CTES: Childhood Traumatic Events Scale ** p < 0.01.

Salience Network: Males with UCPPS exhibited lower betweenness centrality in the right anterior insula (aINS) compared to HC males (*t* = -3.99, *q* = .001) (**Table 4**, **Table 5** and **Fig 3**). The overall model of disease group predicting right aINS betweenness centrality, with CTES scores as the moderator, accounted for 19% of the variance (*F*_(4, 51)_ *=* 3.04, *p* = 0.03, *R*^*2*^ = 0.19). Investigation at the specific levels of EALs revealed that there was a significant moderation effect between disease group and eigenvector centrality in the right aINS at average (*B* = 60.53, *t*_(51)_ = 2.55, *p* = 0.01) and high (*B* = 93.12, *t*_(51)_ = 2.93, *p* = 0.009) values of CTES scores (**Fig 5**).

#### 3.2.3. Females with UCPPS vs. HC females (disease effect within females)

**Fig 6:**
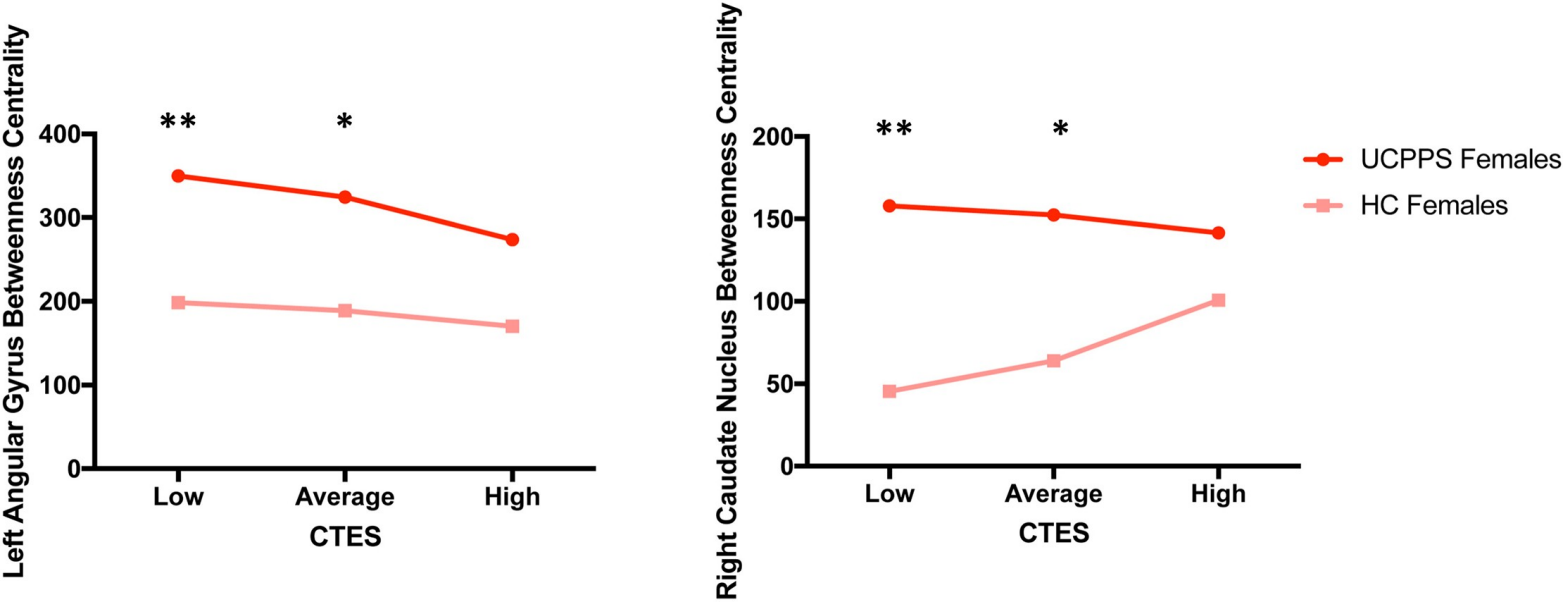
Moderating effect of CTES on the effect of disease within females on centrality of the left angular gyrus (default mode network) and right caudate nucleus (basal ganglia network). Abbreviations: UCPPS: Urologic Chronic Pelvic Pain Syndrome, CTES: Childhood Traumatic Events Scale. *p < 0.05, **p < 0.01.

Basal-Ganglia Network: Females with UCPPS exhibited greater betweenness centrality in the right caudate nucleus (*t* = 3.20, *q* = 0.023) (**Table 4, Table 5, Fig 3**). The overall model of disease group predicting right caudate nucleus betweenness centrality, with CTES scores as the moderator, and age and site as covariates, accounted for 9% of the variance (*F*_(5, 101)_ *=* 1.91, *p* = 0.099, *R*^*2*^ = 0.09), but not significantly. Investigation at the specific levels of EALs revealed that there was a significant moderation effect between sex and betweenness centrality of the right caudate nucleus at both low (*B* = -56.25, *t*_(101)_ = -2.36, *p* = 0.02) and average (*B* = -44.30, *t*_(101)_ = -2.63, *p* = .01) values of CTES scores (**Fig 6**).

Default Mode Network: Females with UCPPS exhibited greater betweenness centrality in the left angular gyrus (*t* = 3.18, *q* = 0.037) (**Table 4, Table 5, Fig 3**). The overall model of disease group predicting left angular gyrus betweenness centrality, with CTES scores as the moderator, and age and site as covariates, significantly accounted for 13% of the variance (*F*_(5, 101)_ *=* 3.11, *p* = 0.01, *R*^*2*^ = 0.13). Investigation at the specific levels of EALs revealed that there was a significant moderation effect between sex and betweenness centrality of the left angular gyrus at both low (*B* = -75.67, *t*_(101)_ = -2.36, *p* = 0.02) and average (*B* = -67.72, *t*_(101)_ = -2.99, *p* = 0.004) values of CTES scores (**Fig 6**).

#### 3.2.4. Males with UCPPS vs. females with UCPPS (sex effect)

**Fig 7:**
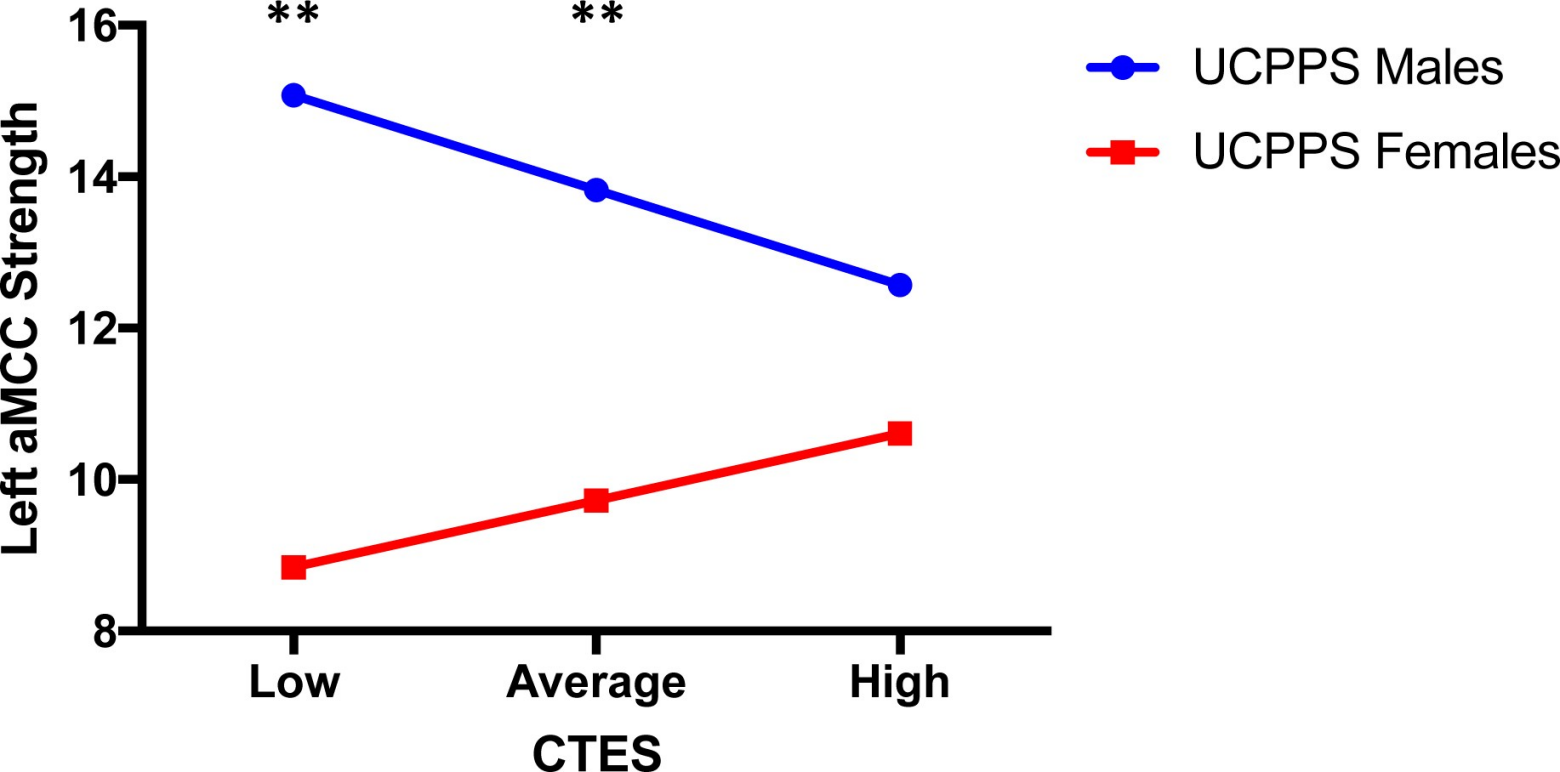
Moderating effect of CTES on the effect of sex within UCPPS on centrality of the left anterior mid-cingulate cortex (salience network). Abbreviations: UCPPS: Urologic Chronic Pelvic Pain Syndrome, CTES: Childhood Traumatic Events Scale, aMCC: anterior mid-cingulate cortex **p < 0.01.

Salience Network: Compared to females, males with UCPPS exhibited greater strength in the left aMCC (MACgG_S) (*t =* 3.14, *q* = 0.03) (**Table 4, Table 5** and **Fig 3**). The overall model of sex predicting left aMCC strength with CTES scores as the moderator accounted for 18% of the variance (*F*_(5, 72)_ *=* 3.08, *p* = 0.01, *R*^*2*^ = 0.18) (**Fig 7**). Investigation at the specific levels of EALs revealed that there was a significant moderation effect between sex and strength of the left aMCC at both low (*B* = -6.23, *t*_(72)_ = -2.70, *p* = 0.009) and average (*B* = -4.10, *t*_(81)_ = -2.66, *p* = 0.01) values of the CTES scores (**Fig 7**).

**Fig 8:**
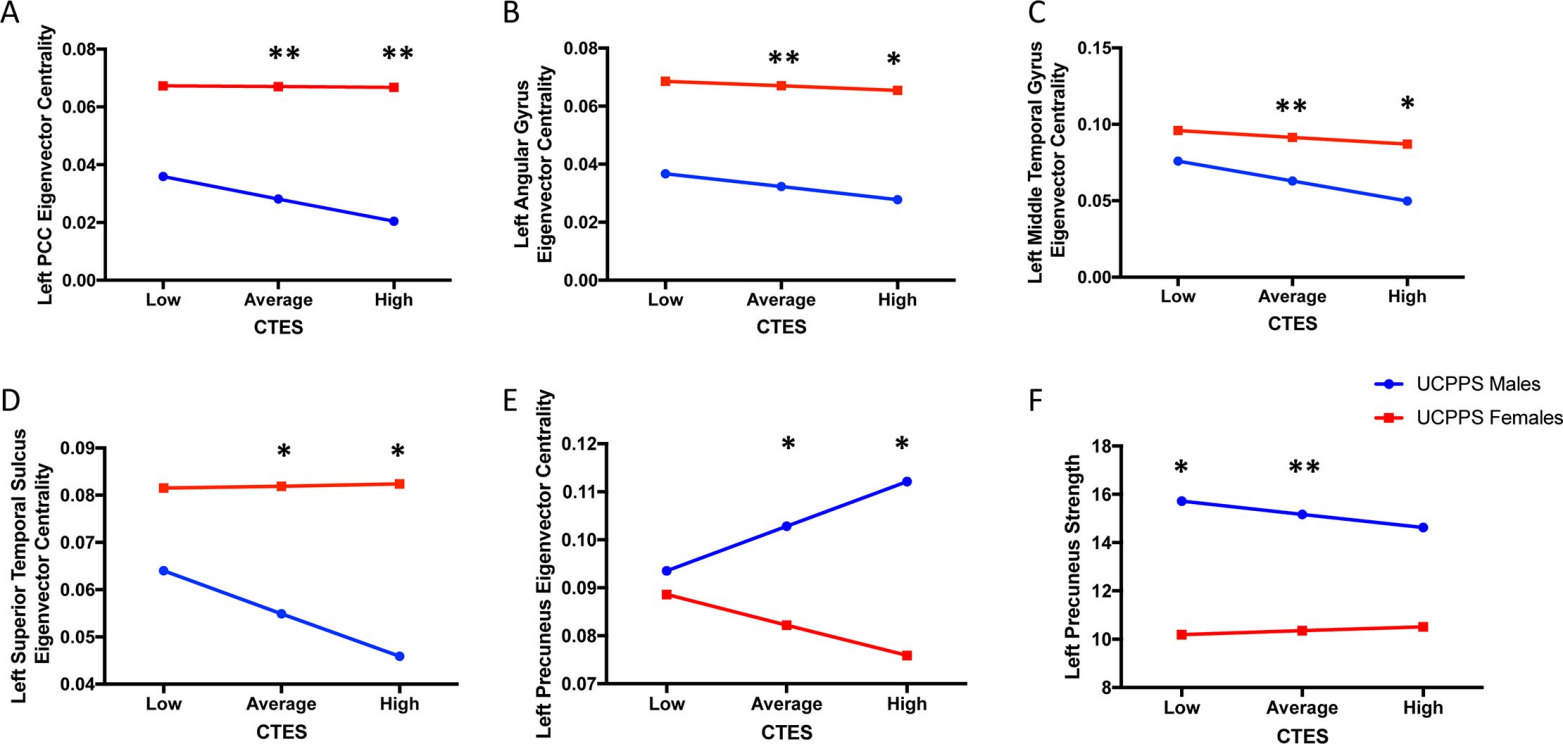
Moderating effect of CTES on the effect of sex within UCPPS on centrality of default mode network regions. Abbreviations: UCPPS: Urologic Chronic Pelvic Pain Syndrome, CTES: Childhood Traumatic Events Scale *p < 0.05, **p < 0.01.

Default Mode Network: Males with UCPPS exhibited lower eigenvector centrality in the left posterior cingulate cortex (PCC) (*t* = -3.10, *q* = .04) (**Table 4, Table 5** and **Fig 3**). The overall model of sex predicting left PCC eigenvector centrality, with CTES scores as the moderator, and age and site as covariates, significantly accounted for 16% of the variance (*F*_(5, 72)_ *=* 2.68, *p* = 0.03, *R*^*2*^ = 0.16). Investigation at the specific levels of EALs revealed that there was a significant moderation effect between sex and eigenvector centrality of the left PCC at both average (*B* = 0.039, *t*_(72)_ = 3.06, *p* = 0.003) and high (*B* = 0.046, *t*_(72)_ = 2.55, *p* = 0.01) values of CTES scores (**Fig 8A**).

Males with UCPPS exhibited lower eigenvector centrality in the left angular gyrus (*t* = -2.81, *q* = .04) (**Table 4, Table 5** and **Fig 3**). The overall model of sex predicting left angular gyrus eigenvector centrality, with CTES scores as the moderator, and age and site as covariates, significantly accounted for 14% of the variance (*F*_(5, 72)_ *=* 2.42, *p* = 0.04, *R*^*2*^ = 0.14). Investigation at the specific levels of EALs revealed that there was a significant moderation effect between sex and eigenvector centrality of the left angular gyrus at both average (*B* = 0.034, *t*_(72)_ = .2.66, *p* = 0.01) and high (*B* = 0.038, *t*_(72)_ = 2.01, *p* = 0.048) values of CTES scores (**Fig 8B**).

Males with UCPPS exhibited lower eigenvector centrality in the left middle temporal gyrus (MTG) (*t* = -2.95, *q* = .04) (**Table 4, Table 5** and **Fig 3**). The overall model of sex predicting left MTG eigenvector centrality, with CTES scores as the moderator, and age and site as covariates, significantly accounted for 17% of the variance (*F*_(5, 72)_ *=* 2.91, *p* = 0.02, *R*^*2*^ = 0.16). Investigation at the specific levels of EALs revealed that there was a significant moderation effect between sex and eigenvector centrality of the left MTG at both average (*B* = 0.029, *t*_(72)_ = 2.65, *p* = 0.01) and high (*B* = 0.037, *t*_(72)_ = 2.41, *p* = 0.02) values of CTES scores (**Fig 8C**).

Males with UCPPS exhibited lower eigenvector centrality in the left superior temporal sulcus (SupTS) (*t* = -2.98, *q* = .04) (**Table 4, Table 5** and **Fig 3**). The overall model of sex predicting left SupTS eigenvector centrality, with CTES scores as the moderator, and age and site as covariates, significantly accounted for 15% of the variance (*F*_(5, 72)_ *=* 2.53, *p* = 0.04, *R*^*2*^ = 0.15). Investigation at the specific levels of EALs revealed that there was a significant moderation effect between sex and eigenvector centrality of the left SupTS at both average (*B* = 0.027, *t*_(72)_ = 2.39, *p* = 0.02) and high (*B* = 0.037, *t*_(72)_ = 2.26, *p* = 0.03) values of CTES scores (**Fig 8D**).

Males with UCPPS exhibited greater eigenvector centrality in the left precuneus compared to females with UCPPS (*t* = 3.25, *q* = 0.03) (**Table 4, Table 5** and **Fig 3**). The overall model of sex predicting left precuneus eigenvector centrality, with CTES scores as the moderator, and age and site as covariates, accounted for 11% of the variance (*F*_(5, 72)_ *=* 1.76, *p* = 0.13, *R*^*2*^ = 0.11), but was not significant. Investigation at the specific levels of EALs revealed that there was a significant moderation effect between sex and eigenvector centrality of the left precuneus at both average (*B* = -0.021, *t*_(72)_ = -2.01, *p* = 0.048) and high (*B* = -0.036, *t*_(72)_ = -2.48, *p* = 0.02) values of CTES scores (**Fig 8E**).

Males with UCPPS exhibited greater strength (*t* = 3.14, *q* = 0.03) in the left precuneus compared to females with UCPPS (**Table 4**, **Table 5** and **Fig 3)**. The overall model of sex predicting left precuneus strength, with CTES scores as the moderator, and age and site as covariates, significantly accounted for 22% of the variance (*F*_(5, 72)_ *=* 4.06, *p* = 0.003, *R*^*2*^ = 0.22). Investigation at the specific levels of EALs revealed that there was a significant moderation effect between sex and strength of the left precuneus at both low (*B* = -5.53, *t*_(72)_ = -2.23, *p* = 0.03) and average (*B* = -4.82, *t*_(72)_ = -2.91, *p* = 0.005) values of CTES scores (**Fig 8F**).

### 3.3. Network metric correlations with symptoms

#### 3.3.1. Males with UCPPS vs. females with UCPPS

Females with UCPPS exhibited positive correlations with strength of the left precuneus (*r*_*(45)*_ = 0.45, *p* = 0.002) and GUPI impact on quality of life scores, as well as positive correlations between strength of the left aMCC (*r*_*(45)*_ = 0.36, *p* = 0.01) and GUPI impact on quality of life scores. No correlations were observed in males with UCPPS. No significant correlations were observed for the other group comparisons (UCPPS vs. HC, Females with UCPPS vs HC Females, and Males with UCPPS vs. HC Males).

## 4. Discussion

We identified group differences in measures of centrality in specific regions associated with UCPPS that are moderated by the presence of self-reported early life trauma. In most cases, this moderation was sex-specific. In our multi-site sample, females with UCPPS reported more EALs than female HCs, but there were no differences in EALs between males with UCPPS and male HCs, a finding consistent with previous analyses within the MAPP Research Network [29]. As previously reported, patients with UCPPS exhibited lower levels of quality of life, and greater pain symptom severity and urinary symptom severity compared to HCs. There was an overall disease-dependent association on measures of centrality with UCPPS patients having lower centrality in the anterior insula compared to HCs. EALs did have a sex-dependent association in the salience network, with greater EALs being associated with lower strength in the anterior mid-cingulate cortex in males with UCPPS, but greater in females with UCPPS. Greater EALs were mostly associated with lower centrality in the default mode network in males (except eigenvector centrality in the precuneus) but showed more nuanced results in females–differing by temporal sulci. This suggests that brain mechanisms in females, even within the default mode network, can contribute specifically to their observed symptoms.

### 4.1. Differences in functional network centrality in patients with UCPPS: The effect of sex and early adversity

Overall, patients with UCPPS had lower resting-state centrality in a region of the salience network–the anterior insula–compared to HCs. The salience network has been implicated in the context of pain [45,64,65] and pain sensitivity [66–69]. The observed lower resting-state centrality in the anterior insula was also present when comparing males with UCPPS with male HCs, but no other contrast, suggesting that males are driving the trend seen when comparing by disease group. Similar findings showing lower resting-state connectivity between regions to and from the anterior insula have also been observed within the MAPP Research Network using ROI-to-ROI-based analyses [16].

When comparing males with UCPPS to females with UCPPS, males with UCPPS exhibited greater centrality in the aMCC, another key node of the salience network. Activity of the anterior insula within the salience network has been shown to mediate stimulus-driven, bottom-up control of attention [70] of behaviorally relevant stimuli, including pain [71]. It also integrates sensory information with emotional and homeostatic relevance [72]. The anterior mid-cingulate cortex (aMCC) has been shown to be involved in fear, prediction of negative consequences and avoidance behaviors in regards to pain processing [73]. These findings suggest changes in the activity of the salience network in patients with UCPPS may have nuanced characteristics, especially in males.

Many more nuanced differences were observed when looking at sex differences within UCPPS. Within the salience network, males with UCPPS had greater centrality in the anterior mid-cingulate cortex (aMCC) compared to females with UCPPS, but the moderating effect of early life trauma on this centrality differed by sex; with early trauma being associated with lower centrality in males, but greater centrality in females. Past research outside the context of chronic pain has shown lower structural centrality in the anterior cingulate in young adults with childhood maltreatment [74], but we only observed this pattern in functional centrality in males with UCPPS, and an opposite trend in females with UCPPS. The increase in centrality of the aMCC in females due to childhood trauma may predispose them to increased perception of pain during a painful crisis, which was supported by a positive association between centrality of the aMCC and greater impact on quality of life in our own sample.

Within the default mode network, males with UCPPS had lower centrality in the angular gyrus and temporal regions compared to females, and the moderating effect of early childhood trauma differed by specific gyri. Specifically, in UCPPS patients, greater early childhood trauma was associated with lower centrality in the middle temporal gyrus in both males and females, but with greater centrality in the superior temporal sulcus in just females. Past research has shown that specifically parental verbal abuse is associated with greater gray matter in the superior temporal gyrus and decreased integrity of the arcuate fasciculus emerging from the superior temporal sulcus [75,76]. It has also been observed that the middle temporal gyrus has lower structural centrality in young adults who were maltreated as children [74], and our results mirror this with regards to functional centrality. Both these regions are well regarded to be key nodes of the default mode network attributing to social cognitive processes, theory of mind (superior temporal sulcus), and self-referential processes (middle temporal gyrus) [77,78]. Perhaps, then the impact of trauma on processes specific to the superior temporal sulcus are buffered in females due to girls and women having a greater ability to engage in theory of mind and social cognitive processes [79,80]. Increased information flow in women through these networks could relate to increased processing of affective components of pain [81], which can result in pain augmentation and reduced quality of life [82].

It was also observed that females with UCPPS compared with female HCs exhibited differences in centrality in regions, specifically—the angular gyrus and caudate nucleus—that were not present in the full sample or in males. The angular gyrus is a key node in the default mode network that has been observed to be more active in rest in a variety of patients with chronic pain, including chronic pelvic pain [21,68,83–85], and is highly associated with interoception and somesthesis [86]. Additionally, females have been shown to have greater activity of the default mode network at rest, including the angular gyrus [87]. The caudate nucleus has also been shown to have a role in chronic pain via endogenous opioid-mediated analgesia, as the striatum is the most densely populated location for opioid receptors in the brain [88–90]. The perception of chronic pain has been related to upregulation of opioid receptors in the caudate nucleus [91]. Overall, women have been shown to have greater opioid receptor availability in the caudate nucleus [92], and to have greater therapeutic efficacy from analgesic medication aimed at opioid receptors [93]. The results observed in females with UCPPS, but not in males with UCPPS provide plausible evidence that these differences may be related to inherent sex differences present in the brain, providing guidance for future research and treatment.

### 4.2. Limitations and directions for future research

As this was a cross-sectional study using retrospective reports of adverse childhood events, it is not possible to determine causality between the observed brain changes in resting-state functional connectivity, early life trauma and UCPPS. Future longitudinal studies are needed to determine if brain differences present in early childhood and adolescence, and the presence of trauma in early childhood, are predisposing factors to the development of UCPPS in adolescence and adulthood. All the observed findings are based on the static connectivity of resting-state networks, and dynamics with or without stimuli are not accounted for. Another limitation is the fact that the study had an unbalanced number of males and females, emphasizing the need to have a similar sample size of males and females in order to get statistically accurate representation of sex-related brain effects. Future studies should a conduct a meta-analysis as the number of imaging studies in patients with UCPPS increases. Finally, as there is no standard atlas to use for parcellating the brain, future research should test the effect of different brain atlases, as the selection of different atlases [94,95] may impact final results. We used a structural atlas due to previous research conducted in patients with UCPPS [16,20,21]. However, there are several disadvantages to using spatial maps to make inferences about functional resting state networks, including that it may average dissimilar functional signals based on an a-priori definition of a region [94,96]. Recently, Arslan et al [97] did a systematic comparison between anatomical, connectivity-driven, and random parcellation methods using resting-state data from the Human Connectome Project. It was shown that functionally driven atlases, such as the Glasser HCP Atlas [98] and Gordon Atlas [99], have greater amounts of reproducibility via measures of homogeneity, parcellation reliability via Silhouette analyses, agreement with task activation using the Bayesian Information Criterion (BIC), and greatest overlap over Brodmann Areas via Dice coefficients, making them more optimal atlases to use in future studies [97]. This would allow a greater degree of precision when assigning regions to specific resting-state networks. However, it is important to note that graph theoretical measures such as network segregation and integration (as used in the current study) can be relatively robust to the underlying parcellation scheme [97].

### 4.3. Conclusions

There is a dearth of reported sex differences in research involving chronic pain, and many studies are heavily imbalanced and include more females than males [45]. We found that patients with UCPPS have disease-related differences, with important moderating influences of sex and early life adversity on resting-state functional connectivity within the salience and default mode networks. These differences are associated with decreased, disease-related quality of life in women, but not men. The findings from this study highlight the need to take into consideration sex as well as multiple psychosocial factors, including a history of childhood trauma in the evaluation of patients with chronic pelvic pain. Furthermore, they emphasize the importance of adopting patient-specific, psychological approaches for treating trauma both early in life and after it has occurred. This may play a key role in preventing and treating central changes involved in the development and persistence of chronic pain [100].

## Supporting information

S1 Table Acquisition parameters. (DOCX)

S2 Table Sensitivity analyses using different thresholds. (DOCX)

S3 Table Motion Parameter Analyses (A) Descriptives of Motion Parameters by Group. (B) Differences between means in framewise displacement and root-mean squared realignment estimates for a priori contrasts. (C) Comparison of analyses of the a-priori contrasts after including framewise displacement and root mean squared realignment estimates as covariates.
(DOCX)

S4 Table Members of the MAPP research network. (DOCX)
