# Supplementary material for: Impact of early adverse life events and sex on functional brain networks in patients with urological chronic pelvic pain syndrome (UCPPS): A MAPP Research Network study
Source: PLoS One. 2019 Jun 20;14(6):e0217610. doi: 10.1371/journal.pone.0217610 (PMC6586272; doi:10.1371/journal.pone.0217610)
Supplement: S1 Table — (DOCX) [file pone.0217610.s001.docx]

**S1 Table: Acquisition Parameters**

| **S1A: 3D Structural MRI Acquisition Parameters** | | | | | |
| --- | --- | --- | --- | --- | --- |
| **Institution name** | **Northwestern** | **UCLA** | **Michigan** | **Stanford** | **UAB** |
| Scanner manufacturer | Siemens | Siemens | Philips | GEMS | Philips |
| Scanner model | Trio Tim | Trio Tim | Ingenia | Discovery MR750 | Achieva |
| Software version | B17 | B15 | 4.1.1–4.1.2 | DV22.0 | 2.6.3 |
| Field strength (T) | 3 | 3 | 3 | 3 | 3 |
| Acquisition type | 3D | 3D | 3D | 3D | 3D |
| Image orientation | Axial obl | Axial obl | Axial obl | Axial obl | Axial obl |
| Flip angle [degrees] | 9 | 9 | 8 | 11 | 9 |
| Repetition time (TR) [ms] | 2200 | 2200 | 6.6–7.1 | 6.8–7.4 | 7.1–7.2 |
| Echo time (TE) [ms] | 3.3 | 3.3 | 4.7 | 2.8 | 3.2–4.7 |
| Inversion time (TI) [ms] | 900 | 900 | 790–850 | 450 | 835–844 |
| Number of averages (NEX) | 2 | 2 | 1 | 2 | 1 |
| Pixel bandwidth [Hz] | 241 | 200 | 246–247 | 391 | 241 |
| Field of view (FOV) [mm] | 256 | 256 | 256 | 220 | 256 |
| Acquisition matrix | 256 × 256 | 256 × 256 | 288 × 288 | 256 × 256 | 288 × 288 |
| Slice thickness | 1 (0) | 1 (0) | 0.9 (0) | 1 (0) | 1 (0) |
| Voxel resolution [mm] | 1 × 1 × 1 | 1 × 1 × 1 | 0.9 × 0.9 × 0.9 | 0.86 × 0.86 × 1 | 1 × 1 × 1 |
| **S1B: 3D Functional MRI Acquisition Parameters** | | | | | |
| **Institution name** | **Northwestern** | **UCLA** | **Michigan** | **Stanford** | **UAB** |
| Scanner manufacturer | Siemens | Siemens | Philips | GEMS | Philips |
| Scanner model | Trio Tim | Trio Tim | Ingenia | Discovery MR750 | Achieva |
| Software version | B17 | B15 | 4.1.1–4.1.2 | DV22.0 | 2.6.3 |
| Field strength (T) | 3 | 3 | 3 | 3 | 3 |
| Acquisition type | 2D EPI | 2D EPI | 2D EPI | 2D EPI | 2D EPI |
| Image orientation | Axial obl | Axial obl | Axial obl | Axial obl | Axial obl |
| Flip angle [degrees] | 77 | 77 | 77 | 77 | 77 |
| Repetition time (TR) [ms] | 2000 | 2000 | 2000 | 2000 | 2000 |
| Echo time (TE) [ms] | 29 | 28 | 30 | 30 | 30 |
| Number of repetitions [frames] | 10,800 | 12,000 | 9000–14,000 | 9600 | 9600 |
| Pixel bandwidth [Hz] | 2003 | 3005 | 2000–2200 | 7813 | 3050 |
| Field of view (FOV) [mm] | 220 | 220 | 220 | 220 | 220 |
| Acquisition matrix | 64 × 64 | 64 × 64 | 64 × 64 | 64 × 64 | 64 × 64 |
| Slice thickness (gap) [mm] | 4 (0.5) | 4 (0.5) | 4 (0.5) | 4 (0.5) | 4 (0.5) |
| Voxel resolution [mm] | 3.44 × 3.44 × 4 | 3.44 × 3.44 × 4 | 3.44 × 3.44 × 4 | 3.44 × 3.44 × 4 | 3.44 × 3.44 × 4 |
