# Supplementary material for: Impact of early adverse life events and sex on functional brain networks in patients with urological chronic pelvic pain syndrome (UCPPS): A MAPP Research Network study
Source: PLoS One. 2019 Jun 20;14(6):e0217610. doi: 10.1371/journal.pone.0217610 (PMC6586272; doi:10.1371/journal.pone.0217610)
Supplement: S2 Table — (DOCX) [file pone.0217610.s002.docx]

**S2 Table: Sensitivity Analyses using Different Thresholds**

| **UCPPS vs HC** | | | | | | | | |
| --- | --- | --- | --- | --- | --- | --- | --- | --- |
| Network | Region of Interest | Network Metric | Z = 0.3 | Z = 0.4 | Z = 0.5 | Z = 0.6 | Z = 0.7 | Z = 0.8 |
| Salience Network | Right Anterior Insula (InfCirIns) | Betweeness Centrality | X |  |  | X |  |  |
| **UCPPS Males vs. UCPPS Females** | | | | | | | | |
| Network | Region of Interest | Network Metric | Z = 0.3 | Z = 0.4 | Z = 0.5 | Z = 0.6 | Z = 0.7 | Z = 0.8 |
| Default Mode Network | Left Posterior Cingulate Cortex (PosDCgG) | Eigenvector Centrality | X | X | X |  |  |  |
|  | Left Angular Gyrus | Eigenvector Centrality | X | X | X | X |  |  |
|  | Left Middle Temporal Gyrus | Eigenvector Centrality | X | X | X |  |  |  |
|  | Left Superior Temporal Sulcus | Eigenvector Centrality | X | X | X |  |  |  |
|  | Left Precuneus (CgSMarp) | Eigenvector Centrality | X |  |  | X | X |  |
|  | Left Precuneus (CgSMarp) | Strength | X | X | X | X | X | X |
| Salience Network | Left aMCC | Strength | X | X | X | X |  |  |
| **UCPPS Males vs. HC Males** | | | | | | | | |
| Network | Region of Interest | Network Metric | Z = 0.3 | Z = 0.4 | Z = 0.5 | Z = 0.6 | Z = 0.7 | Z = 0.8 |
| Salience Network | Right Anterior Insula (InfCirIns) | Betweeness Centrality | X | X | X | X |  |  |
| **UCPPS Females vs. HC Females** | | | | | | | | |
| Network | Region of Interest | Network Metric | Z = 0.3 | Z = 0.4 | Z = 0.5 | Z = 0.6 | Z = 0.7 | Z = 0.8 |
| Basal Ganglia | Right Caudate Nucleus | Betweeness Centrality | X | X |  |  |  |  |
| Default Mode Network | Left Angular Gyrus | Betweeness Centrality | X | X |  |  |  |  |

Groups: UCPPS: Urological chronic pelvic pain syndrome, HC: Healthy controls

Regions: InfCirIns: Inferior segment of the circular sulcus of the insula, PosDCgG: Posterior dorsal part of the cingulate gyrus, CGSMarp: Marginal branch of the cingulate sulcus, aMCC: Anterior mid-cingulate cortex

An “X” signifies that there were significant differences between patients with UCPPS and HCs in network metrics when single subjects network were thresholded at different Z thresholds

**Results from the sensitivity analysis for all four contrasts reveal that a threshold at Z = 0.3 is the most sensitive and provides the most robust results.**
