# Supplementary material for: Impact of early adverse life events and sex on functional brain networks in patients with urological chronic pelvic pain syndrome (UCPPS): A MAPP Research Network study
Source: PLoS One. 2019 Jun 20;14(6):e0217610. doi: 10.1371/journal.pone.0217610 (PMC6586272; doi:10.1371/journal.pone.0217610)
Supplement: S3 Table — Motion Parameter Analyses (A) Descriptives of Motion Parameters by Group. (B) Differences between means in framewise displacement and root-mean squared realignment estimates for a priori contrasts. (C) Comparison of analyses of the a-priori contrasts after including framewise displacement and root mean squared realignment estimates as covariates. (DOCX) [file pone.0217610.s003.docx]

**S3 Table: Motion Parameter Analyses**

**A: Descriptives of Motion Parameters by Group**

| Group | Average FD | | | Average RMS | | |
| --- | --- | --- | --- | --- | --- | --- |
|  | Mean | SD | N | Mean | SD | N |
| UCPPS | 0.2089195 | 0.1141226 | 78 | 0.04907307 | 0.0273881 | 78 |
| HC | 0.1956552 | 0.1101048 | 84 | 0.04520363 | 0.02707008 | 84 |
| UCPPS Males | 0.2110674 | 0.10706858 | 29 | 0.05040268 | 0.02719667 | 29 |
| UCPPS Females | 0.2076482 | 0.11916814 | 49 | 0.04828616 | 0.02775123 | 49 |
| HC Males | 0.1623911 | 0.07295938 | 26 | 0.03715099 | 0.01802145 | 26 |
| HC Females | 0.2105667 | 0.12077765 | 58 | 0.04881343 | 0.02969447 | 58 |

**Abbreviations:** FD: framewise displacement, RMS: root-mean squared realignment estimates, UCPPS: urologic chronic pelvic pain syndrome, HC: healthy controls, SD: standard deviation, N: number of subjects

**B: Differences between means in framewise displacement and root-mean squared realignment estimates for a priori contrasts**

| Contrast | Variable | *t* | *df* | *p* |
| --- | --- | --- | --- | --- |
| UCPPS vs. HC | FD | 0.748 | 160 | 0.4527 |
|  | RMS | 0.9039 | 160 | 0.3694 |
| UCPPS Males vs. UCPPS Females | FD | 0.1271 | 29 | 0.8992 |
|  | RMS | 0.3279 | 76 | 0.7239 |
| UCPPS Males vs. HC Males | FD | 1.9472 | 53 | 0.0568 |
|  | RMS | 2.1037 | 53 | 0.0402 |
| UCPPS Females vs. HC Females | FD | 0.1253 | 105 | 0.9005 |
|  | RMS | 0.0943 | 105 | 0.9251 |

**Abbreviations:** FD: framewise displacement, RMS: root-mean squared realignment estimates, UCPPS: urologic chronic pelvic pain syndrome, HC: healthy controls, t: t-value, df: degrees of freedom, p: p-value

**Results show that there are no significant differences in motion parameters between groups for the a priori contrasts, except UCPPS males vs. HC Males regarding RMS.**

**C: Comparison of analyses of the a-priori contrasts after including framewise displacement and root mean squared realignment estimates as covariates**

| UCPPS vs. HC | | | | | | |
| --- | --- | --- | --- | --- | --- | --- |
| Original Analysis | | | | | | |
|  | *beta* | *se* | *t* | *p* | *q* | *df* |
| NodeBWCent__R_CaN | 29.81781557 | 11.11582539 | 2.68246527 | *0.027291506* | 0.109166 | 153 |
| NodeBWCent__L_AngG | 40.5893435 | 15.94092868 | 2.546234558 | *0.039319706* | 0.2359 | 153 |
| NodeBWCent__R_InfCirIns | -44.00071649 | 13.96171084 | -3.151527559 | *0.006989178* | *0.02795671* | 153 |
| RMS | | | | | | |
| NodeBWCent__R_CaN | 28.68116693 | 11.2674945 | 2.545478671 | *0.038951966* | 0.1558079 | 152 |
| NodeBWCent__L_AngG | 40.6310752 | 16.18175568 | 2.510918841 | *0.042939181* | 0.2576351 | 152 |
| NodeBWCent__R_InfCirIns | -44.37904524 | 14.17121584 | -3.131632863 | *0.00740526* | *0.0222157* | 152 |
| FD | | | | | | |
| NodeBWCent__R_CaN | 28.70010902 | 11.24012682 | 2.553361673 | *0.038780191* | 0.1551208 | 152 |
| NodeBWCent__L_AngG | 40.15577524 | 16.14483359 | 2.487221377 | *0.045894061* | 0.2753644 | 152 |
| NodeBWCent__R_InfCirIns | -44.42865771 | 14.13982061 | -3.142094864 | *0.007158041* | *0.02147412* | 152 |
| UCPPS M vs. HC M | | | | | | |
| Original Analysis | | | | | | |
|  | *beta* | *se* | *t* | *p* | *q* | *df* |
| NodeBWCent__R_InfCirIns | -181.470599 | 45.45009892 | -3.992743763 | *0.000320936* | *0.001283744* | 153 |
| RMS | | | | | | |
| NodeBWCent__R_InfCirIns | -182.912452 | 46.32967246 | -3.948062697 | *0.000945356* | *0.003781424* | 152 |
| FD | | | | | | |
| NodeBWCent__R_InfCirIns | -183.097613 | 46.18824608 | -3.964160324 | *0.000385069* | *0.001540276* | 152 |
| UCPPS F vs. HCF | | | | | | |
| Original Analysis | | | | | | |
|  | *beta* | *se* | *t* | *p* | *q* | *df* |
| NodeBWCent__R_CaN | 83.61412108 | 26.09292557 | 3.204474748 | *0.005690978* | *0.02276391* | 153 |
| NodeBWCent__L_AngG | 119.0045012 | 37.41921548 | 3.180304548 | *0.00621097* | *0.037* | 153 |
| NodeBWCent__R_LoInG_CInS | -76.90188485 | 26.58137317 | -2.893074198 | *0.01458116* | 0.102 | 153 |
| RMS | | | | | | |
| NodeBWCent__R_CaN | 83.39942015 | 26.14286859 | 3.190140357 | *0.006155734* | *0.02462294* | 152 |
| NodeBWCent__L_AngG | 119.0123838 | 37.54494953 | 3.169863999 | *0.006229057* | *0.03737434* | 152 |
| NodeBWCent__R_LoInG_CInS | -77.15846135 | 26.61776939 | -2.898757602 | *0.014520009* | 0.1016401 | 152 |
| FD | | | | | | |
| NodeBWCent__R_CaN | 83.39276577 | 26.13555032 | 3.190779025 | *0.006097955* | *0.02439182* | 152 |
| NodeBWCent__L_AngG | 118.9186355 | 37.53997776 | 3.167786518 | *0.006451245* | *0.03870747* | 152 |
| NodeBWCent__R_LoInG_CInS | -77.21650763 | 26.58145683 | -2.904901268 | *0.014277088* | 0.09993962 | 152 |
| UCPPS M vs. UCPPS F | | | | | | |
| Original Analysis | | | | | | |
|  | *beta* | *se* | *t* | *p* | *q* | *df* |
| EigenvecCent__L_PosDCgG | -0.036724401 | 0.011860033 | -3.096483813 | *0.007958984* | *0.037928* | 153 |
| EigenvecCent__L_AngG | -0.033214185 | 0.011811068 | -2.812123843 | *0.01881969* | *0.04234* | 153 |
| EigenvecCent__L_MTG | -0.033558329 | 0.011377286 | -2.949590085 | *0.012642934* | *0.0379288* | 153 |
| EigenvecCent__L_CgSMarp | 0.029048459 | 0.010644315 | 2.729011531 | *0.023662239* | *0.04259* | 153 |
| EigenvecCent__L_SupTS | -0.034317367 | 0.011511932 | -2.981025965 | *0.011838794* | *0.037928* | 153 |
| EigenvecCent__R_PosDCgG | -0.036195281 | 0.012027282 | -3.009431478 | *0.010706013* | 0.06423608 | 153 |
| EigenvecCent__R_MTG | -0.033117918 | 0.012118013 | -2.732949684 | *0.023806527* | 0.1428392 | 153 |
| NodeBWCent__L_SupTS | -142.2966642 | 62.65047402 | -2.271278333 | 0.077412739 | 0.4644764 | 153 |
| Strength__L_MACgG_S | 4.840225562 | 1.542639961 | 3.137624906 | *0.007408317* | *0.0296* | 153 |
| Strength__L_CgSMarp | 5.675184648 | 1.745279033 | 3.251734846 | *0.005053708* | *0.04548337* | 153 |
| Strength__R_MACgG_S | 3.771296981 | 1.48063338 | 2.547083587 | *0.039265274* | 0.1177958 | 153 |
| RMS | | | | | | |
| EigenvecCent__L_PosDCgG | -0.036131885 | 0.011977155 | -3.016733588 | *0.01066914* | *0.03983945* | 152 |
| EigenvecCent__L_AngG | -0.035555828 | 0.011827085 | -3.00630538 | *0.010756857* | *0.0323067* | 152 |
| EigenvecCent__L_MTG | -0.034561753 | 0.011475781 | -3.011712523 | *0.010241657* | *0.0323067* | 152 |
| EigenvecCent__L_CgSMarp | 0.02872343 | 0.010753266 | 2.671135426 | *0.028518312* | 0.0501162 | 152 |
| EigenvecCent__L_SupTS | -0.036665737 | 0.011521368 | -3.182411826 | *0.006084311* | *0.03650587* | 152 |
| EigenvecCent__R_PosDCgG | -0.034235457 | 0.012079192 | -2.83425051 | *0.017867048* | 0.1072023 | 152 |
| EigenvecCent__R_MTG | -0.033222931 | 0.01224444 | -2.713307394 | *0.025422886* | 0.1525373 | 152 |
| NodeBWCent__L_SupTS | -156.3250702 | 62.57751224 | -2.498102986 | *0.044676067* | 0.2680564 | 152 |
| Strength__L_MACgG_S | 4.858802667 | 1.558709569 | 3.117195637 | *0.007447983* | *0.02979193* | 152 |
| Strength__L_CgSMarp | 5.70969538 | 1.763360635 | 3.237962369 | *0.004990762* | *0.04491686* | 152 |
| Strength__R_MACgG_S | 3.957173202 | 1.490722239 | 2.654534223 | *0.029649017* | 0.08894705 | 152 |
| FD | | | | | | |
| EigenvecCent__L_PosDCgG | -0.035930167 | 0.011948711 | -3.007032963 | *0.010544384* | *0.03936792* | 152 |
| EigenvecCent__L_AngG | -0.035243251 | 0.011810786 | -2.983988684 | *0.011704511* | *0.03511353* | 152 |
| EigenvecCent__L_MTG | -0.034402374 | 0.011458891 | -3.002242839 | *0.011078249* | *0.03511353* | 152 |
| EigenvecCent__L_CgSMarp | 0.028939259 | 0.010737812 | 2.695079625 | *0.026553823* | *0.04779688* | 152 |
| EigenvecCent__L_SupTS | -0.036431013 | 0.011497111 | -3.168710187 | *0.006765576* | *0.04059346* | 152 |
| EigenvecCent__R_PosDCgG | -0.034107114 | 0.012024713 | -2.83641816 | *0.017496854* | 0.1049811 | 152 |
| EigenvecCent__R_MTG | -0.033202817 | 0.012224657 | -2.716053131 | *0.024849746* | 0.1490985 | 152 |
| NodeBWCent__L_SupTS | -154.3055461 | 62.51259921 | -2.468391141 | *0.047759139* | 0.2865548 | 152 |
| Strength__L_MACgG_S | 4.880848129 | 1.555919502 | 3.136954144 | *0.007089907* | *0.02835963* | 152 |
| Strength__L_CgSMarp | 5.684501662 | 1.760649034 | 3.228639866 | *0.005495116* | *0.04945604* | 152 |
| Strength__R_MACgG_S | 3.938455243 | 1.488047514 | 2.646726806 | *0.029803885* | 0.08941165 | 152 |

**Abbreviations:** FD: framewise displacement, RMS: root-mean squared realignment estimates, UCPPS: urologic chronic pelvic pain syndrome, HC: healthy controls, M: male, F: female, t: t-value, se: standard error, df: degrees of freedom, p: p-value, q: FDR-corrected p-value, NodeBWCent: node betweenness centrality, EigenvecCent: eigenvector centrality, L: Left, R:right, PosCDgG: dorsal posterior cingulate cortex, AngG: angular gyrus, MTG: middle temporal gyrus, CGSMarp: marginal part of the cingulate sulcus, SupTS: superior temporal sulcus, MACgG_S: anterior mid-cingulate cortex, CaN: caudate nucleus, LoInG_CInS: long insular gyrus and sulcus, InfCirIns: inferior segment of the circular sulcus of the insula. Italicized values represent significant values.

**Results show that including RMS and FD as covariates did not influence results, thus motion was not a serious confounding variable when comparing a-priori contrasts.**
