# Supplementary material for: Impact of early adverse life events and sex on functional brain networks in patients with urological chronic pelvic pain syndrome (UCPPS): A MAPP Research Network study
Source: PLoS One. 2019 Jun 20;14(6):e0217610. doi: 10.1371/journal.pone.0217610 (PMC6586272; doi:10.1371/journal.pone.0217610)
Supplement: S4 Table — (DOCX) [file pone.0217610.s004.docx]

**S4 Table: Members of the MAPP Research Network**

| **Site** | Role(s) | Personnel | |
| --- | --- | --- | --- |
|  | MAPP Network Executive Committee | J. Quentin Clemens, MD, FACS, MSci, Network Chair, 2013-  Philip Hanno, MD  Ziya Kirkali, MD  John W. Kusek, PhD | J. Richard Landis, PhD  M. Scott Lucia, MD  Chris Mullins, PhD  Michel A. Pontari, MD |
| Northwestern University | Discovery Site | David J. Klumpp, PhD, Co-Director  Anthony J. Schaeffer, MD, Co-Director  Apkar (Vania) Apkarian, PhD  David Cella, PhD  Melissa A. Farmer, PhD  Colleen Fitzgerald, MD  Richard Gershon, PhD  James W. Griffith, PhD | Charles J. Heckman II, PhD  Mingchen Jiang, PhD  Laurie Keefer, PhD  Darlene S. Marko, RN, BSN, CCRC  Jean Michniewicz  Todd Parrish, PhD  Frank Tu, MD, MPH |
| University of California, Los Angeles | Discovery Site, Neuroimaging Core | Emeran A. Mayer, MD, Co-Director  Larissa V. Rodríguez, MD, Co-Director  Jeffry Alger, PhD  Cody P. Ashe-McNalley  Ben Ellingson, PhD  Nuwanthi Heendeniya | Lisa Kilpatrick, PhD  Jason Kutch, PhD  Jennifer S. Labus, PhD  Bruce D. Naliboff, PhD  Fornessa Randal  Suzanne R. Smith, RN, NP |
| University of Iowa | Discovery Site | Karl J. Kreder, MD, MBA, Director  Catherine S. Bradley, MD, MSCE  Mary Eno, RN  Kris Greiner, BA | Yi Luo, PhD, MD  Susan K. Lutgendorf, PhD  Michael A. O’Donnell, MD  Barbara Ziegler, BA |
| University of Michigan | Discovery Site | Daniel J. Clauw, MD, Co-Director; Network Chair, 2008-2013  J. Quentin Clemens, MD, FACS, MSci, Co-Director; Network Chair, 2013-  Suzie As-Sanie, MD  Sandra Berry, MA  Megan E. Halvorson, BS, CCRP | Richard Harris, PhD  Steve Harte, PhD  Eric Ichesco, BS  Ann Oldendorf, MD  Katherine A. Scott, RN, BSN  David A. Williams, PhD |
| University of Washington, Seattle | Discovery Site | Dedra Buchwald, MD, Director  Niloofar Afari, PhD, Univ. Of California, San Diego  John Krieger, MD  Jane Miller, MD  Stephanie Richey, BS | Susan O. Ross, RN, MN  Roberta Spiro, MS  TJ Sundsvold, MPH  Eric Strachan, PhD  Claire C. Yang, MD |
| Washington University, St. Louis | Discovery Site | Gerald L. Andriole, MD, Co-Director  H. Henry Lai, MD, Co-Director  Rebecca L. Bristol, BA, BS  Graham Colditz, MD, DrPH  Georg Deutsch, PhD, Univ. of  Alabama at Birmingham  Vivien C. Gardner, RN, BSN  Robert W. Gereau IV, PhD  Jeffrey P Henderson, MD, PhD | Barry A. Hong, PhD, FAACP  Thomas M. Hooton, MD, Univ of Miami  Timothy J. Ness, MD, PhD, Univ. of Alabama at Birmingham  Carol S. North, MD, MPE, Univ.  Texas Southwestern  Theresa M. Spitznagle, PT, DPT, WCS  Siobhan Sutcliffe, PhD, ScM, MHS |
